# Artificial intelligence adoption in French cardiovascular care: a multiprofessional survey of barriers and facilitators

**DOI:** 10.1093/ehjdh/ztag042

**Published:** 2026-04-17

**Authors:** Nabil Bouali, Séverine Domart, Walid Amara, Christophe Laure, Floran Begue, Johann Reisberg, Thierry Garban, Guillaume Bailly, Simon Viscogliosi, Chaimae Aboueddahab, Soundous M’Rabet, Sébastien Hascoet, Guillaume Baudry, Manveer Singh, Youssef Lakhal, Paul Lucain, Gauthier Beuque, Philippe Régnier, Antonin Trimaille, Marc Villaceque, Orianne Weizman, Jérémie Barraud, Stéphane Lafitte, Cyril Ferdynus, Louis-Marie Desroche

**Affiliations:** Cardiology Department, University Hospital of Poitiers, 2 rue de la Milétrie, CS 90577, Poitiers CEDEX 86021, France; Department of Cardiology, University Hospital of La Réunion - Saint-Denis, North Site CS 11021, Allée des Topazes, 97400 Saint-Denis, La Réunion, France; Rhythmology Unit, Intermunicipal Hospital Group Le Raincy Montfermeil, Montfermeil, France; Clinical Research and Innovation Directorate, Chartres Hospital Center, Le Coudray, France; USMD, Clinical Research and Innovation Unit (DRCI), University Hospital of La Réunion, Saint-Pierre, France; Cardiology Department, Pitié-Salpêtrière Hospital, Assistance Publique–Hôpitaux de Paris (AP-HP), Paris, France; Department of Cardiology, National Union of Cardiologists, Paris, France; Cardiology Department, Saint-Antoine and Tenon Hospitals, AP-HP, Sorbonne University, Paris, France; Sorbonne University, AP-HP, Inserm CIC-1901, Department of Pharmacology, Pitié-Salpêtrière Hospital, Paris, France; Department of Cardiology, Hospices Civils de Lyon, Lyon, France; Cardiology Department, Ibn Sina University Hospital, Mohammed V University of Rabat, Rabat, Morocco; Department of Cardiovascular Medicine, Nouvel Hôpital Civil, Strasbourg University Hospital, Strasbourg, France; Cardiology Department, Dijon University Hospital, Dijon, France; Pediatric and Congenital Cardiology Working Group (FCPC), French Society of Cardiology, Paris, France; Department of Congenital Heart Diseases, Marie Lannelongue Hospital, M3C Network, Le Plessis-Robinson, France; Department of Medicine, Paris-Saclay University, Kremlin-Bicêtre School of Medicine, Le Kremlin-Bicêtre, Paris, France; Hypertension and Therapeutic Innovation Unit, INSERM UMR-S 999, Le Plessis-Robinson, France; Université de Lorraine, Inserm CIC-1433, Inserm U1116, Nancy University Hospital, Nancy, France; INI-CRCT (Cardiovascular and Renal Clinical Trialists), F-CRIN Network, Nancy, France; REICATRA, Université de Lorraine, Vandœuvre-lès-Nancy, France; Cardiology Department, Hôpital Lariboisière, AP-HP, Paris, France; Faculty of Medicine, Université Paris Cité, Paris, France; Cardiology Department, Amiens University Hospital, Amiens, France; Cardiology Department, Amiens University Hospital, Amiens, France; Cardiology Department, Bordeaux University Hospital, Bordeaux, France; Cardiology Department, Paul d’Égine Private Hospital, Champigny-sur-Marne, France; Department of Cardiovascular Medicine, Strasbourg University Hospital, Strasbourg, France; Private Practice, Cabinet de Cardiologie Libérale, Nîmes, France; Cardiology Department, Clinique Claude Bernard, Metz, France; Cardiology Department, Aix-en-Provence Hospital, Aix-en-Provence, France; Cardiology Department, Bordeaux University Hospital, Bordeaux, France; CIC, Centre d'Investigation Clinique - Epidémiologie Clinique (CIC-EC) University Hospital of La Réunion - Saint-Denis, North Site CS 11021, Allée des Topazes, 97400 Saint-Denis, La Réunion, France; Department of Cardiology, University Hospital of La Réunion - Saint-Denis, North Site CS 11021, Allée des Topazes, 97400 Saint-Denis, La Réunion, France; CIC, Centre d'Investigation Clinique - Epidémiologie Clinique (CIC-EC) University Hospital of La Réunion - Saint-Denis, North Site CS 11021, Allée des Topazes, 97400 Saint-Denis, La Réunion, France

**Keywords:** Artificial intelligence, Cardiology, Digital health, Adoption, Explainable AI, Training

## Abstract

**Aims:**

Responsible adoption of artificial intelligence (AI) in cardiology remains uneven. We aimed to map knowledge, attitudes, beliefs and practices among cardiovascular professionals in France and to identify levers for implementation.

**Methods and results:**

We conducted a national multiprofessional survey across cardiovascular care from 4 December 2024 to 1 March 2025. Prespecified outcomes included regular use in practice, confidence in diagnostic outputs, performance expectations, training needs, and social influence. Seven hundred fifty-six professionals completed the survey (58.2% cardiologists, 24.3% allied-health professionals, 17.8% other professionals; median age 37 years; 46.7% women). AI use was reported as regular (≥ weekly) by 23%, occasionally by 40%, and none by 37%; only 7.8% had formal AI training. Use concentrated on AI-assisted imaging (32%) and patient monitoring/management (18%). The most valued benefit was improved diagnostic accuracy (29%); leading concerns were algorithmic bias (29.9%) and data privacy (28.2%). Explainability increased confidence (among cardiologists, high confidence 64% in therapeutic contexts vs. 84% with explanations). In multivariable analyses, prior training (aOR 3.22, 95% CI 1.60–6.55), research involvement (2.94, 1.90–4.58), and male sex (1.64, 1.05–2.59) were associated with higher use, while age > 40 years was associated with lower use (0.62, 0.40–0.96). Allied-health professionals reported lower social influence and training needs.

**Conclusion:**

Adoption of AI in cardiology remains limited, and four levers emerged for responsible scale-up: Training (education), Explainability (transparent outputs), Integration (workflow embedding), and Accompaniment (peer support, evaluation). These priorities should guide education, governance, and procurement strategies.

## Graphical Abstract

**Graphical Abstract:**
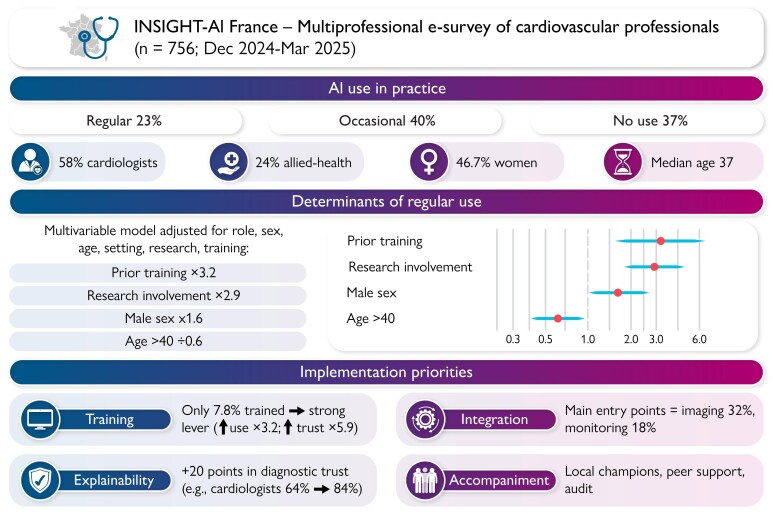

## Introduction

Artificial intelligence (AI) is expanding rapidly in cardiology, with cardiovascular publications growing by 20–25% annually since 2016,^1^ and a widening portfolio of validated applications in imaging, intervention, and clinical decision support, including early experiences with large language models (LLMs).^2–4^ Yet translation into daily practice remains uneven, with persistent gaps between technological advances and real-world adoption.^5^ Recognising this challenge, the European Society of Cardiology (ESC) has made AI and digital health central to its 2023–2028 strategic plan to foster safe and equitable integration across Europe.^6^

AI is no longer confined to data scientists: machine learning, deep learning, and LLMs are increasingly embedded in clinical software and workflows.^2–4^ This diffusion fuels enthusiasm but also blurs conceptual boundaries,^4^ while raising expectations for earlier detection, improved diagnostic accuracy, and efficiency gains.^2,5^ However, successful deployment requires more than accuracy metrics. It depends on aligning professional competencies, workflow integration, and trustworthy-use frameworks,^5,7,8^ at a time when the European AI Act introduces new requirements for transparency, accountability, and governance in clinical applications.^9,10^ Despite progress, prospective real-world validation in cardiology remains incomplete.^11^

To date, no multiprofessional mapping of knowledge, attitudes, beliefs, and practices (KABP) regarding AI in cardiology has been published in Europe. The INSIGHT-AI France survey addresses this gap by providing a multiprofessional assessment of KABP and identifying key barriers and facilitators to clinical adoption.

## Methods

### Study design and reporting framework

INSIGHT-AI France was designed as a France-wide, cross-sectional, multi-stakeholder survey to assess knowledge, attitudes, beliefs and practices (KABP) regarding barriers and facilitators to AI adoption among cardiovascular professionals. The survey followed CHERRIES (Checklist for Reporting Results of Internet E-Surveys) standards,^12^ to ensure methodological rigour, transparency, and reproducibility (see Supplementary material online, Table  *S1*). A 12-month follow-up is planned as an exploratory extension to capture temporal dynamics, in line with the fast-evolving nature of AI. The rationale and full methodological protocol have already been reported.^13^

### Population and recruitment

The survey was designed to capture the full spectrum of cardiovascular professionals, including cardiologists at all career stages, allied-health professionals specialized in cardiology, institutional leaders, and non-clinical digital health actors (e.g. biomedical engineers, IT managers, data scientists). Recruitment covered all healthcare settings, from academic hospitals to private practices and institutional structures. Exclusion criteria were non-cardiologist physicians, non-specialized allied-health professionals or students, respondents outside the French healthcare ecosystem, age <18 years, and lack of consent.

Recruitment was conducted nationwide between 4 December 2024 and 1 March 2025, through a multi-channel dissemination strategy (societies, subspecialty groups, newsletters, social media, QR codes, congress presentations, institutional relays). Engagement was reinforced by a network of regional ambassadors. To reduce selection bias and enhance representativeness, a stratified, non-proportional quota design ensured balanced inclusion across institution type, professional role, and subspecialty. Baseline characteristics were compared between respondents who completed the questionnaire and those who did not, to assess potential completion bias (see Supplementary material online, Table  *S2*)

### Data governance, protection and transparency

Scientific oversight was provided by the Collège des Cardiologues en Formation (CCF; National College of Cardiologists-in-Training) and the Cercle Cardio-AI (AI Working Group) of the French Society of Cardiology, with endorsement from affiliated subspecialty groups. This governance structure ensured broad representation across academic, hospital-based, and private practice cardiology.

Ethical approval was obtained from a national Health Research Ethics Committee (approval number withheld for double-blind review). Participation was voluntary, with mandatory electronic informed consent required before the first survey item. Data were captured via the GDPR-compliant SKEZIA platform, hosted on EU-based servers with French health-data hosting certification. The platform automatically pseudonymised the responses, with strict separation of identifiers from survey data. A four-step authentication procedure prevented duplicate entries without compromising anonymity.

### Questionnaire development

The questionnaire was developed through a structured consensus process and overseen by a multidisciplinary scientific committee. To ensure representativeness, the draft instrument was circulated for review across all subspecialty groups of the French Society of Cardiology. The final version comprised a 20-item KABP-based core section (Knowledge, Attitudes, Beliefs, Practices) supplemented by profession-specific modules. Question formats included multiple-choice, binary, Likert-scale, and ranking items. Structured across seven online pages with skip logic, the survey required <15 min to complete on average. A pilot test (*n* = 20) improved clarity and usability prior to launch. The full questionnaire is available in the published protocol.^13^

### Outcomes and statistical analysis

Within a KABP framework for cardiology, we prespecified five practice-focused endpoints to characterize AI adoption in routine care: regular AI use in clinical practice (at least weekly) encompassing both AI functionalities embedded within clinical software when identified as such by respondents and more visible AI tools used by professionals, acknowledging that some embedded components may not always be explicitly perceived as AI by users; high diagnostic trust in AI outputs, defined by a Likert-based trust score with a prespecified high-trust cut-point > 60/100, consistent with OECD guidelines^14^; high performance expectations, defined as a requirement that AI performance exceed human-expert level before adoption; a perceived need for additional AI training (yes/no); and perceived social influence from peers and supervisors. Social influence was defined based on respondents’ self-reported conditions for AI adoption in clinical practice and distinguished (i) peer-related influence (adoption if colleagues use AI successfully), (ii) institutional or scientific validation (adoption if supported by clinical guidelines or scientific evidence), (iii) individual pioneering behaviour, and (iv) scepticism toward AI.

Respondents also completed preference rankings for perceived benefits, barriers, facilitators and ethical concerns to prioritize implementation levers.

Analyses were conducted in R (v4.4.2). Continuous variables were summarized as mean ± SD when visually compatible with normality (histograms) or median [IQR] otherwise; categorical variables as *n* (%). Group comparisons used Student’s *t*-test or Mann–Whitney U tests for continuous variables, χ^2^ or Fisher tests for categorical variables, and one-way ANOVA for multi-group comparisons where appropriate. All analyses were conducted on an available-case basis; the analytic sample size is reported for each table/figure when necessary. Preference rankings (benefits, barriers, facilitators, ethical concerns) were modelled using Plackett–Luce (Plackett-Luce). Five multivariable logistic regressions were fitted for the prespecified outcomes (regular AI use, high diagnostic trust, high performance expectations, perceived need for additional training, perceived social influence), reporting adjusted odds ratios (aOR) with 95% CIs. Covariates included prior AI training, professional role (cardiologist vs. allied-health professionals), research involvement, sex, university-hospital practice, and age > 40 years (threshold previously applied to distinguish digital-native vs. digital-immigrant groups).^15^ High performance expectations were defined as selecting either ‘no error tolerated’ or ‘performance superior to a medical expert’. Variance-inflation factors were assessed for all models. Two-sided *P* < 0.05 defined statistical significance. To further assess the potential confounding effect of age, sensitivity analyses were performed by comparing logistic regression models with and without age adjustment. Relative changes in regression coefficients were examined in the Supplementary material online, Table  *S3*.

## Results

### Study population and professional roles

**Figure 1:**
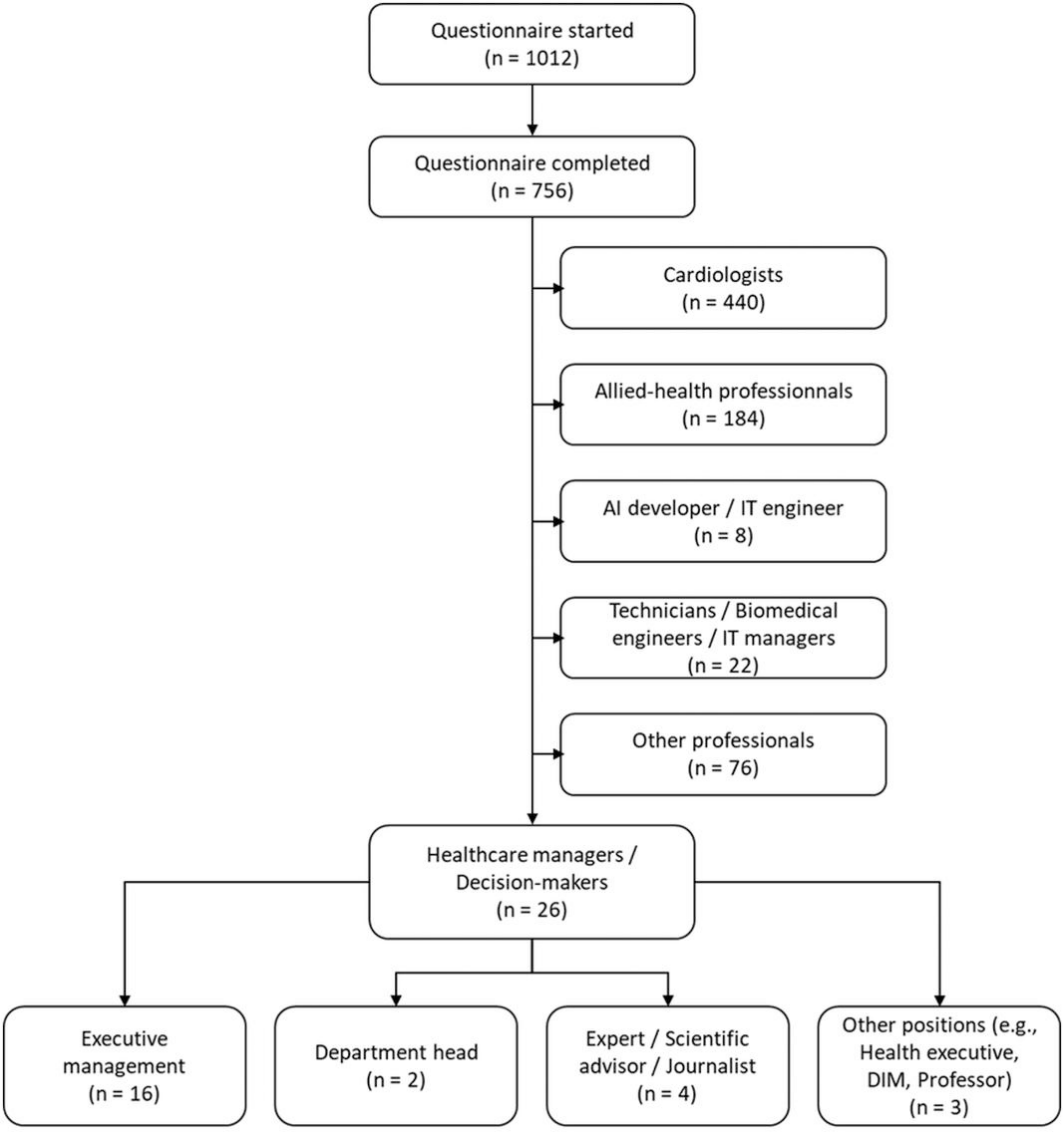
**Flowchart of survey participants**. Participant disposition from questionnaire start to completion and final professional categories; the healthcare manager/decision-maker sub-group is detailed. **Abbreviations:** IT, information technology.

Between 4 December 2024 and 1 March 2025, a total of 756 healthcare professionals completed the survey, with representation from all French regions, including overseas territories (see Supplementary material online, Figure  *S1*). The cohort had a balanced sex distribution (46.7% women) and a median age of 37 years [IQR 30–46], with 40.3% aged 40 years or older (*Table 1*). Age-stratified sociodemographic characteristics, training and attitudes are detailed in Supplementary material online, Table  *S4*, and the distribution of AI use across age groups is shown in Supplementary material online, Figure  *S2*. Cardiologists represented 58.2% of respondents (*n* = 440), allied-health professionals 24.3% (*n* = 184), and the remainder comprised AI developers, policy stakeholders, engineers, IT managers, and other professionals; overall study flow is shown in *Figure 1*. Half of the participants practised in university hospitals (49.8%), followed by general hospitals (27.0%), private hospitals or clinics (15.4%), and private cardiology practices (15.4%) (Respondents could report more than one practice setting). Within the physician group, the most common subspecialties were general cardiology (41.5%), interventional cardiology (21.7%) and electrophysiology (17.9%) (see Supplementary material online, Table  *S5*); allied-health roles are presented in Supplementary material online, Table  *S6*.

**Table 1:** Baseline characteristics of survey participants

	Overall (*n* = 756)
**Sex, *n* (%)**	
Men	403 (53.3%)
Women	353 (46.7%)
**Age (years), median [IQR]**	37 [30; 46]
**Age > 40 years, *n* (%)**	305 (40.3%)
**Professional status, *n* (%)**	
Cardiologist	440 (58.2%)
Allied-health professionals	184 (24.3%)
Health AI developer	8 (1.1%)
Health policy decision-maker	26 (3.4%)
Technician, engineer, or IT manager	22 (2.9%)
Other	76 (10.1%)
**Type of workplace, *n* (%)** **(multiple responses)**	
University hospital (CHU)	376 (49.8%)
General hospital or ESPIC	204 (27%)
Private hospital or clinic	116 (15.4%)
Private cardiology practice	116 (15.4%)
Multidisciplinary health centre	14 (1.9%)
Company or startup	29 (3.8%)
Institutional structure	7 (0.9%)
Other	9 (1.2%)
Missing data	1 (0.1%)
**Use of AI, *n* (%)**	
No use	272 (37.2%)
Occasional use	287 (39.3%)
Regular use	172 (23.5%)
Missing data	25 (3.5%)
**Trained in AI, *n* (%)**	57 (7.8%)
Missing data	25 (3.5%)

Values are expressed as a number (percentage) unless otherwise indicated. IQR: interquartile range. AI: artificial intelligence. CHU: Centre Hospitalier Universitaire (University hospital). ESPIC: Établissement de Santé Privé d’Intérêt Collectif (non-profit private hospital).

### Exposure to and training in artificial intelligence

**Figure 2:**
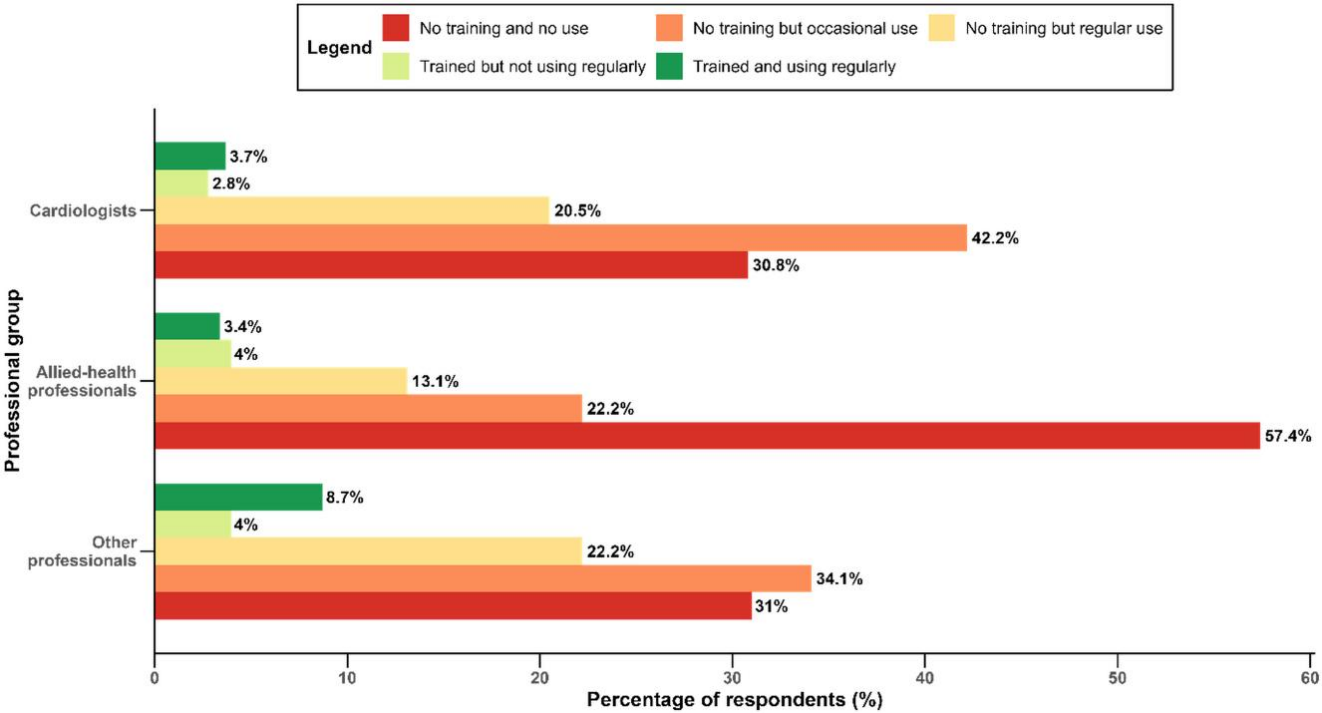
**Exposure to and training in AI according to professional group**. Stacked bars showing joint categories of AI training and use across professional groups. Group differences were assessed using a Pearson chi-square test of independence (*P* < 0.001). Categories: no training & no use; no training & occasional use; no training & regular use; trained & not using regularly; trained & using regularly. Regular use: ≥ weekly. **Abbreviations:** AI, artificial intelligence.

Overall, 57 participants (7.8%) reported having received formal training in AI. Regular AI use, defined as at least weekly, was reported by 23% of respondents, occasional use by 40%, and no use by 37% (*Table 2* and *Figure 2*). Cardiologists were over-represented among occasional users, while allied-health professionals were more often non-users. Regular use was reported by 58% of those with prior AI training vs. 21% of those without. AI use varied across age groups, with lower proportions of regular use among the youngest (<30 years) and oldest (≥60 years) respondents, and the highest proportion of regular AI users observed in the 50–59-year age group. Detailed multivariable associations, including the independent effects of training, age, and sex, are presented in the ‘Multivariable analysis’ section below.

**Table 2:** Exposure to and training in artificial intelligence

	Overall (*n* = 731)	No AI use (*n* = 272)	Occasional AI use (*n* = 287)	Regular AI use (*n* = 172)	*P*-value
**Demographics**					
Age, years—median [IQR]	37 [30; 45]	36 [28; 44]	37 [30; 45]	37.5 [30; 47]	*P* = 0.357
Age > 40 yrs	284 (38.9%)	98 (36%)	113 (39.4%)	73 (42.4%)	*P* = 0.391
Women	343 (46.9%)	156 (57.4%)	118 (41.1%)	69 (40.1%)	*P* < 0.001
**Professional categories**					
Cardiologists	429 (58.7%)	132 (48.5%)	193 (67.2%)	104 (60.5%)	*P* < 0.001
Allied-health professionals^a^	176 (24.1%)	101 (37.1%)	46 (16%)	29 (16.9%)	
AI developer/IT-Engineer	8 (1.1%)	0 (0%)	0 (0%)	8 (4.7%)	
Policy/Management stakeholder	22 (3%)	3 (1.1%)	11 (3.8%)	8 (4.7%)	
Technician, engineer, or IT manager	22 (3%)	6 (2.2%)	10 (3.5%)	6 (3.5%)	
Other professionals	74 (10.1%)	30 (11%)	27 (9.4%)	17 (9.9%)	
**Workplace**					
University hospital	365 (50%)	138 (50.7%)	143 (50%)	84 (48.8%)	*P* = 0.927
Non-university hospital/clinic^b^	305 (41.8%)	117 (43%)	119 (41.6%)	69 (40.1%)	*P* = 0.831
Other (start-up, regulator, etc.)	43 (5.9%)	8 (2.9%)	14 (4.9%)	21 (12.2%)	*P* < 0.001
Missing data, *n*(%)	1 (0.1%)	0 (0%)	1 (0.3%)	0 (0%)	
**Training & attitudes**					
Formal AI training	57 (7.8%)	0 (0%)	24 (8.4%)	33 (19.2%)	*P* < 0.001
No perceived need for training	90 (12.4%)	61 (22.6%)	16 (5.6%)	13 (7.6%)	*P* < 0.001
Missing data, *n*(%)	3 (0.4%)	2 (0.7%)	1 (0.3%)	0 (0%)	
Would adopt if colleagues succeed (Yes)	616 (84.6%)	238 (88.1%)	248 (86.7%)	130 (75.6%)	*P* < 0.001
Missing data, *n*(%)	3 (0.4%)	2 (0.7%)	1 (0.3%)	0 (0%)	
**Professional roles (multiple responses)**					
Clinical care	572 (94.5%)	218 (93.6%)	228 (95.4%)	126 (94.7%)	*P* = 0.676
Research	140 (23.1%)	28 (12%)	61 (25.5%)	51 (38.3%)	*P* < 0.001
Teaching/communication	151 (25%)	26 (11.2%)	76 (31.8%)	49 (36.8%)	*P* < 0.001
Administrative/organisational duties	126 (20.8%)	31 (13.3%)	56 (23.4%)	39 (29.3%)	*P* < 0.001
Other roles	19 (3.1%)	4 (1.7%)	7 (2.9%)	8 (6%)	*P* = 0.074
Missing data, *n*(%)	126 (17.2%)	39 (14.3%)	48 (16.7%)	39 (22.7%)	

The analytic sample size for this table is 731 participants, corresponding to respondents who provided complete data on AI use frequency. Values are expressed as a number (percentage) unless otherwise indicated. IQR: interquartile range. AI: artificial intelligence.

^a^Allied-health professionals include nurses, radiology technicians, and related staff.

^b^Non-university hospitals include general hospitals, private hospitals, and clinics. Missing data are shown where applicable. *P* values correspond to χ^2^ tests or Kruskal–Wallis tests as appropriate.

### Perceived benefits, risks, barriers and facilitators

**Figure 3:**
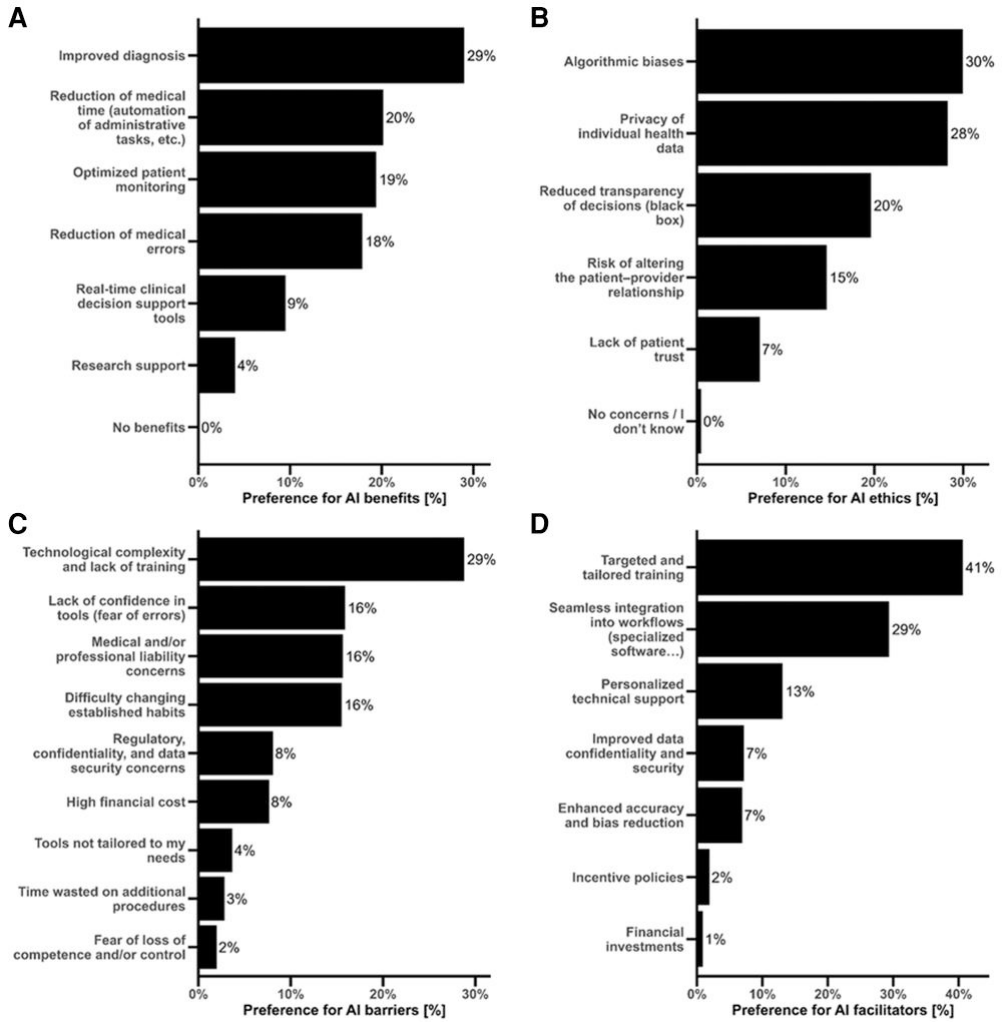
**Ranked perceptions of AI benefits, ethical concerns, barriers, and facilitators**. Panels A–D display preference probabilities from Plackett–Luce models of ranking questions for (*A*) benefits, (*B*) ethical concerns, (*C*) barriers, and (*D*) facilitators. Bars indicate the estimated probability of each item being ranked highest (95% CIs shown). **Abbreviations:** AI, artificial intelligence; CI, confidence interval.

Preference-ranking analysis revealed a consistent and statistically significant hierarchy across all domains (*P* < 0.01, *Figure 3*). Improvement of diagnostic accuracy was considered the most important benefit (29%), while support for research was rarely prioritized (4%). The main ethical concerns were algorithmic bias (29.9%) and data privacy (28.2%), whereas loss of patient trust was ranked lowest (7%). The leading perceived barrier was technological complexity combined with lack of training (29%), while regulatory and financial constraints were assigned much lower and non-consensual probabilities. The most important facilitators were targeted training programmes (40.6%) and seamless integration into existing workflows (29.3%), whereas financial or policy incentives were consistently ranked as least influential (<2%).

### Current AI tools and chatbot use

AI-assisted image analysis was the most frequently used tool (32% overall, including 47% of cardiologists), followed by patient monitoring or management systems (18%). Clinical predictive modelling (9%) and personalized care algorithms (3%) were rarely implemented. Forty-four percent of participants reported no use of AI tools, with allied-health professionals most frequently represented in this group (61% vs. 34% of cardiologists, *P* < 0.001) (see Supplementary material online, Table  *S7*).

Chatbot use differed significantly across professions (*P* < 0.01; see Supplementary material online, Figure  *S3* for frequency patterns and Supplementary Figure  *S4* for usage domains; detailed counts in Supplementary material online, Table  *S8*). Cardiologists reported the widest range of applications, including research activities (25%), teaching or communication (23%), and clinical practice (21%). Allied-health professionals were more likely to declare no use of chatbots (42% vs. 28% of cardiologists and 17% of other professionals). Non-clinical professionals, such as engineers and policymakers, primarily use chatbots for administrative and research purposes.

### Performance expectations

Acceptable performance thresholds varied across professional groups (see Supplementary material online, Figure  *S5*). Nearly half of cardiologists (48%) and other professionals (48%) considered AI acceptable if comparable to the average clinician, whereas this proportion was lower among allied-health professionals (35%). A substantial share of allied-health professionals required AI to perform at least at an expert level (21%) or beyond expert level (21%), compared with 28% and 7% respectively among cardiologists, and 30% and 5% among other professionals. Between 7% and 13% of respondents across groups selected ‘not applicable’ or ‘other’.

### Confidence in AI

**Figure 4:**
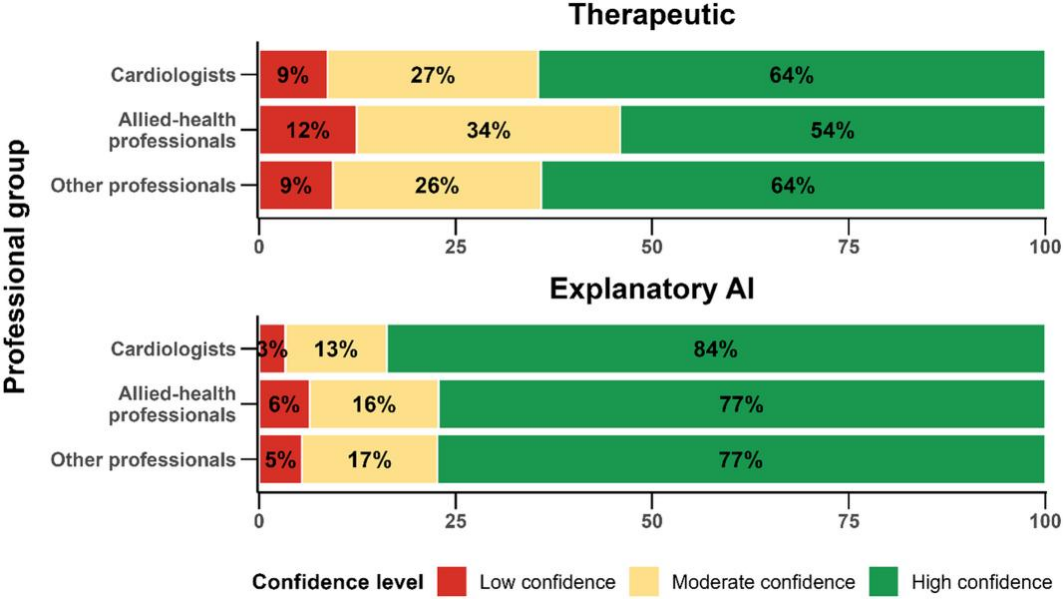
**Confidence in AI by clinical context (therapeutic vs. explanatory)**. Stacked bars of confidence levels in AI outputs by context (therapeutic decision-support; explanatory AI) and professional group. Confidence bins: low (0–30), moderate (31–60), high (61–100). **Abbreviations:** AI, artificial intelligence.

Confidence levels in AI outputs differed by clinical context but not significantly by professional group when analysed as distributions (*P* = 0.224 for therapeutic, *P* = 0.220 for explanatory AI; *Figure 4*). These descriptive findings are complemented by multivariable analyses presented later in this section, which explore determinants of high confidence (>60/100). For therapeutic decision-making, high confidence was reported by 64% of cardiologists, 54% of allied-health professionals, and 64% of other professionals. Confidence increased markedly when the AI provided an explanatory rationale, reaching 84% among cardiologists and 77% among both allied health and other professionals.

### Training needs

**Figure 5:**
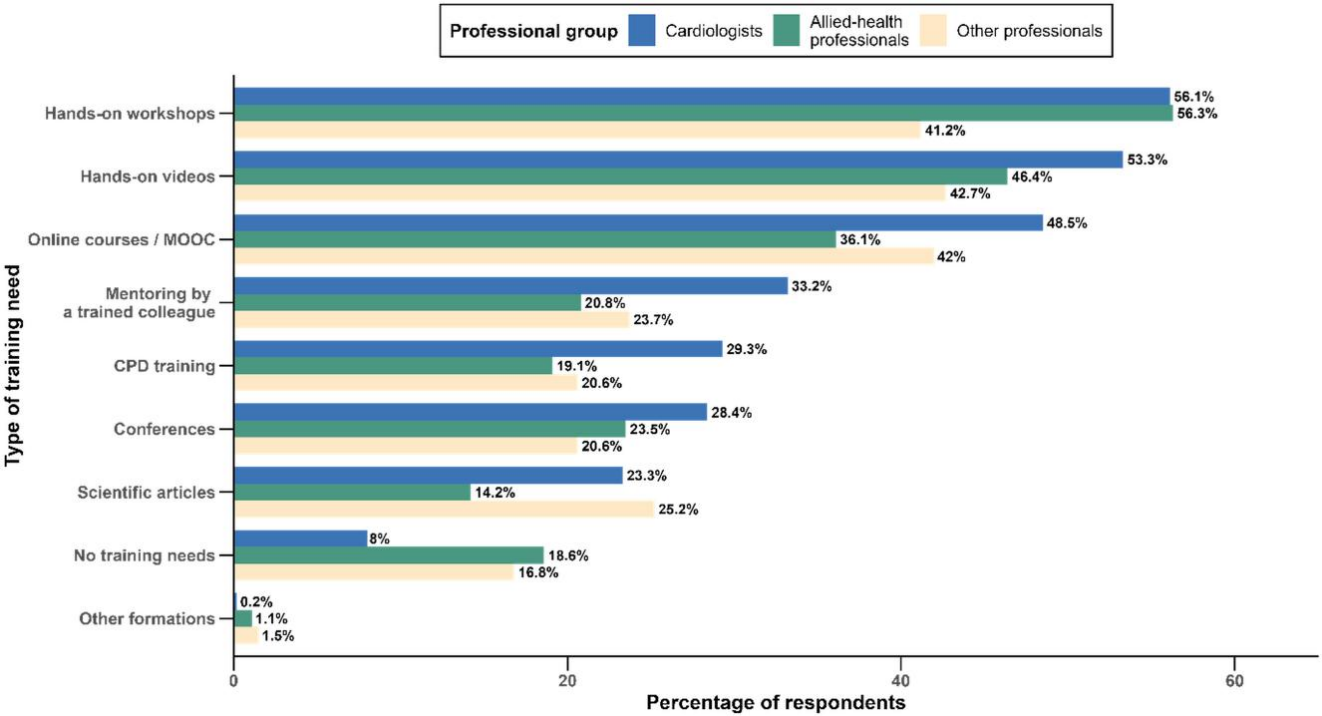
**Training needs and preferred learning formats across professional groups**. Bar plot of preferred AI training formats by professional group: hands-on workshops/face-to-face courses, hands-on videos, online courses/MOOC, mentoring by a trained colleague, CPD training, conferences, scientific articles, other, and no training needed. **Abbreviations:** AI, artificial intelligence; CPD, continuing professional development; MOOC, massive open online course.

Training preferences varied across professional groups. Hands-on workshops or face-to-face courses were the most frequently selected option (53.5% overall), with similar uptake among cardiologists (56.1%) and allied-health professionals (56.3%), but lower among other professionals (41.2%, *P* = 0.006). Online courses or MOOCs (44.3% overall) were more often chosen by cardiologists (48.5%) than by allied-health professionals (36.1%) or others (42.0%, *P* < 0.05). Conversely, declaring no training need was significantly more common among allied-health professionals (18.6%) and other professionals (16.8%) compared with cardiologists (8.0%, *P* < 0.001) (*Figure 5*, Supplementary material online, Table  *S9*).

### Multivariable analysis

**Figure 6:**
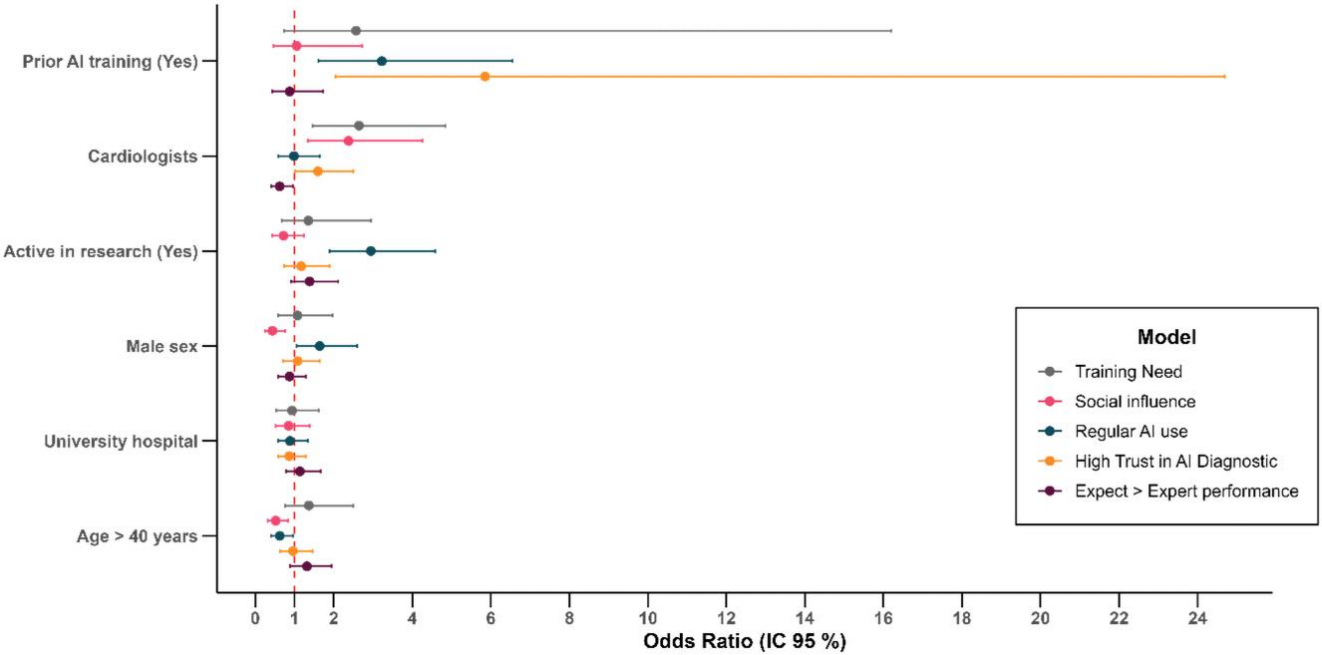
**Multivariable determinants of AI adoption and perceptions (forest plots)**. Forest plot of adjusted odds ratios from five logistic models for: expectation of performance superior to a medical expert; high trust in diagnostic AI; regular AI use (≥ weekly); social-influence–driven adoption; and reporting an AI training need. Covariates in all models: prior AI training, cardiologist status, active research involvement, sex, university-hospital practice, and age > 40 years. Vertical line denotes aOR = 1; whiskers indicate 95% CIs. **Abbreviations:** AI, artificial intelligence; aOR, adjusted odds ratio; CI, confidence interval.

Multivariate logistic regression identified several consistent determinants of AI adoption and perceptions (*Figure 6*, Supplementary material online, Table  *S10*). Regular AI use was associated with prior AI training (aOR 3.22, 1.60–6.55, *P* = 0.001), research involvement (aOR 2.94, 1.90–4.58, *P* < 0.001) and male sex (aOR 1.64, 1.05–2.59, *P* = 0.031), and was inhibited by age > 40 years (aOR 0.62, 0.40–0.96, *P* = 0.04). High diagnostic trust was independently associated with prior training (aOR 5.85, 2.04–24.69, *P* = 0.004) and was more frequent among cardiologists than allied-health professionals (aOR 1.59, 1.02–2.50, *P* = 0.04). Expectation of performance superior to an expert was less common among cardiologists than among allied-health professionals (aOR 0.62, 0.41–0.96, *P* = 0.03). Adoption under social influence was significantly more frequent among cardiologists compared with allied-health professionals (aOR 2.37, 1.33–4.26, *P* < 0.01), but less likely in men (aOR 0.44, 0.25–0.76, *P* = 0.004) and in respondents aged > 40 years (aOR 0.52, 0.32–0.84, *P* = 0.008). No significant association was observed with prior training, research activity, or practice setting. Finally, cardiologists were more likely than allied-health professionals to report a training need (aOR 2.64, 1.45–4.85, *P* = 0.002). Variance-inflation factors were < 2 for all models, indicating the absence of relevant multicollinearity. Effect estimates were similar across models with and without age adjustment, with sensitivity analyses showing only minimal relative changes in regression coefficients (see Supplementary material online, Table  *S3*).

## Discussion

### Key findings

In our cohort (*n* = 756), 23% reported weekly AI use, 37% reported no use, and only 7.8% had formal training; prior training was strongly associated with regular use (aOR 3.22, 95% CI 1.60–6.55) and high diagnostic trust (aOR 5.85, 2.04–24.69), while research involvement also favoured regular use (aOR 2.94, 1.90–4.58) and age > 40 years was associated with lower regular use (aOR 0.62, 0.40–0.96). Consistent with the ranking analysis, respondents identified two primary facilitators—targeted training and seamless workflow integration (preference probabilities 40.6% and 29.3%). Explainability raised trust levels, with cardiologists’ confidence increasing from 64% to 84%, and about 77% among allied-health and other professionals. Social influence was more salient among younger professionals and women. In practice, this influence was driven mainly by institutional and scientific validation rather than peer imitation, with 60% of respondents reporting adoption conditional on guideline endorsement, compared with 25% conditional on successful peer use (see Supplementary material online, Table  *S7*). Usage concentrated in imaging (32% overall; 47% among cardiologists) and monitoring/management (18%), reflecting immediate clinical entry points.

### Barriers and facilitators

Barriers centred on complexity and lack of training; the most cited ethical concerns were algorithmic bias (29.9%) and data privacy (28.2%). Training and workflow integration emerged as the most influential facilitators. Taken together, these findings translate into four levers: Train, Explain, Integrate, and Accompany. ‘Accompany’ emphasizes the importance of local champions, peer mentoring, and structured evaluation to sustain adoption, especially among allied-health professionals, older professionals, and subgroups reporting lower uptake.

### Practical implications

The survey highlights four immediate priorities for responsible adoption of AI in cardiology. First (i), education remains the most powerful lever: role-specific curricula, short online modules, bedside workshops and near-peer mentoring should be scaled, as even minimal training was strongly associated with increased use and diagnostic trust.^16^ Content should be tailored, for example, with imaging interpretation, bias appraisal and governance for cardiologists, and hands-on exposure for allied-health professionals in workflow-heavy settings such as telemonitoring, catheterisation laboratories or intensive care. Conceptually, AI should act as a cognitive exoskeleton, strengthening clinicians’ perception, reasoning and action. Training and deployment must therefore prioritize augmentation, not replacement, of clinical judgement.^17^

Second (ii), explainability should be embedded by default at the point of decision, since clinicians reported substantial gains in confidence when systems provided transparent rationales.^2,18^ Explainability must be actionable and aligned with clinical reasoning rather than purely technical outputs.

Third (iii), integration into daily workflows is essential. Integrating AI into existing imaging and monitoring workflows means connecting it directly to the hospital’s usual systems—such as the Picture Archiving and Communication System (PACS) and DICOMweb imaging standards, or the electronic health record (EHR) via the Fast Healthcare Interoperability Resources (FHIR) protocol.^19–22^ If these tools also provide single sign-on access (one login for all applications) and maintain audit trails (automatic records of who did what and when), they both match the facilitators most valued by clinicians and help address concerns around patient privacy and algorithmic bias. Our data indicate that embedded AI tools in imaging (32%) and monitoring (18%) currently represent the main clinical entry points, whereas chatbot use in clinical practice (∼16%; 21% among cardiologists) remains modest and should be accompanied by clear safeguards on traceability, confidentiality and human oversight.^23,24^ In contrast, non-clinical chatbot applications (research, teaching, administrative support) were more frequently reported (see Supplementary material online, Figure  *S3*–*SS4* and *Table S8*).

Fourth (iv), successful deployment requires sustained accompaniment, through local champions, peer endorsement and structured pre/post evaluation. This is particularly important given the adoption lag among allied-health professionals, who more often reported no AI use (61% vs. 34% of cardiologists) and no chatbot exposure (42% vs. 28%), and who expressed higher performance thresholds and less declared training need.^25^ Without adequate support, untrained or poorly informed use carries risks of over-reliance on outputs, misinterpretation of probabilities, or propagation of bias, underscoring the need for a human-in-the-loop approach.^26^

In practical terms, respondents’ priorities can be summarized into four procurement criteria: explainability by default, systematic bias audits, traceability with SSO and audit trails, and plug-and-play interoperability with PACS and EHR systems.^9,10,22^ These directions also align with the ESC 2023 –2028 strategic pillars of education, digital capacity and equitable care.^6^

### Comparison with prior work

Our results echo the international ‘maturity gap’: rapid algorithmic progress but limited bedside integration.^5,27^ In European surveys, lack of structured training—not enthusiasm—emerges as the dominant barrier.^7,8^ Systematic reviews confirm that facilitators cluster around training and workflow integration, while complexity and governance concerns remain key obstacles.^25^ The independent association between male sex and AI use may reflect a combination of contextual and professional factors, including differential exposure to AI-enabled environments, greater representation in technology-intensive cardiology subspecialties where AI tools are more frequently implemented, and broader structural disparities in Science, Technology, Engineering, and Mathematics (STEM) domains,^28^ as described in the ESC Atlas of Cardiology.^29^

In our cohort, training not only increased exposure but also co-varied with trust, suggesting that education helps clinicians understand limitations and calibrate expectations. The strong influence of explainability on trust aligns with broader calls for transparent model behaviour and human-centred design.^18,30^ Ethical concerns around bias and privacy mirror those highlighted in global policy documents.^31–33^ Compared with earlier European snapshots and reviews, our study adds national multiprofessional coverage with adjusted estimates for adoption and diagnostic trust, prioritizes barriers and facilitators via ranking rather than unweighted lists, and quantifies the explainability (trust increment in clinical contexts).

### Strengths and limitations

Strengths of this work include its large multiprofessional scope across the French cardiovascular ecosystem, adherence to CHERRIES standards (see Supplementary material online, Table  *S1*), use of preference ranking models to prioritize barriers and facilitators, and multivariable analyses adjusting for role and demographic factors. Limitations include the cross-sectional, self-reported design; voluntary participation with non-proportional quotas; and the absence of population weighting, which limits representativeness. The relatively young median age reflects selection mechanisms related to our digital recruitment strategy, which may have favoured more digitally engaged professionals. While this limits generalisability regarding absolute adoption rates, the main barriers and facilitators remained robust after age adjustment in multivariable analyses.

It is worth noting that 440 cardiologists and cardiology residents (approximately 6% of the national cardiology community) participated. This is another measure of coverage, rather than representativeness.^34^ Our broad definition of AI captured real-world practices but blended heterogeneous tools, reflecting the current state of AI deployment in cardiovascular care. In routine practice, this heterogeneity is partly driven by a dichotomy between AI components that are seamlessly embedded within clinical software and workflows and may not always be explicitly perceived as AI by users, and AI tools that are clearly labelled, promoted, or recognized as such within dedicated applications. This coexistence mirrors the rapid diffusion and growing enthusiasm surrounding AI implementation and highlights the challenges of defining AI use along strict technical boundaries in real-world settings. Importantly, these AI modalities are associated with distinct risk–benefit profiles: embedded diagnostic tools are primarily designed to enhance precision but may be associated with automation bias, whereas generative models such as large language models are more frequently associated with issues related to hallucinations, reliability, and data privacy.

Finally, the secure identification workflow (email code verification; ∼1-minute setup) may have constrained respondent numbers but prevented duplicate entries and enabled longitudinal follow-up for consenting participants. Prior AI training was associated with higher adoption, but confidence intervals were wide, and this result should be interpreted with caution.

### Future directions

The planned twelve-month follow-up will capture temporal trajectories of adoption, confidence and training impact, and will allow mixed-effects modelling of change. As this cross-sectional analysis provides a baseline assessment of AI adoption, qualitative approaches could complement the follow-up by contextualising individual attitudes and adoption mechanisms over time and across subgroups.

Hybrid effectiveness–implementation studies should assess the impact of AI deployment on diagnostic timeliness, error prevention, workflow burden and equity across subgroups.^16,35,36^ Qualitative studies will be essential to elucidate mechanisms of social influence, particularly among younger professionals and women.^25^ To strengthen comparability and decision relevance, future research should also apply international standards for AI trials such as CONSORT-AI and SPIRIT-AI, and predefine core outcome sets aligned with procurement criteria.^5,35,36^

## Conclusion

INSIGHT-AI France isolates four decisive levers for responsible AI in cardiology: Training, Explainability, Integration, and Accompaniment. Each is directly grounded in our data: very low baseline training, large gains in trust from explainability, high priority placed on integration, and subgroup-specific adoption gaps. Anchoring deployment in education, transparent governance, standards-based integration, and structured mentoring can help move European cardiology from early curiosity to confident, patient-centred use at scale.

## Supplementary Material

ztag042_Supplementary_Data

## Data Availability

De-identified data and R code underlying this article will be available from the corresponding author upon reasonable request, under a data-use agreement.

## Lead author biography

Louis-Marie Desroche, MD, is an interventional cardiologist at the University Hospital of La Réunion and associate researcher at the Clinical Investigation Centre (INSERM 1410). His work focuses on efficient pathways in ischaemic cardiomyopathy (PHRC-*n* CAMAREC), minimalist catheter-based management of pulmonary embolism (SUEVA-EKOS), and explainable AI as a ‘cognitive exoskeleton’ to support decision-making. He co-ordinates regional and national initiatives (REC, INSIGHT-AI France, AIDOPT) and founded the École Numérique de Cardiologie to translate evidence into practice and education. Dr Desroche serves as Deputy Editor for the European Journal of Preventive Cardiology and leads several multicentre collaborations from La Réunion.
