# Supplementary material for: Artificial intelligence adoption in French cardiovascular care: a multiprofessional survey of barriers and facilitators
Source: Eur Heart J Digit Health. 2026 Apr 17;7(4):ztag042. doi: 10.1093/ehjdh/ztag042 (PMC13131982; doi:10.1093/ehjdh/ztag042)
Supplement: ztag042_Supplementary_Data [file ztag042_supplementary_data.zip › Revised_Supplementary_For_Review.docx]

# Supplemental Data

## **Supplementary eFigures**

**
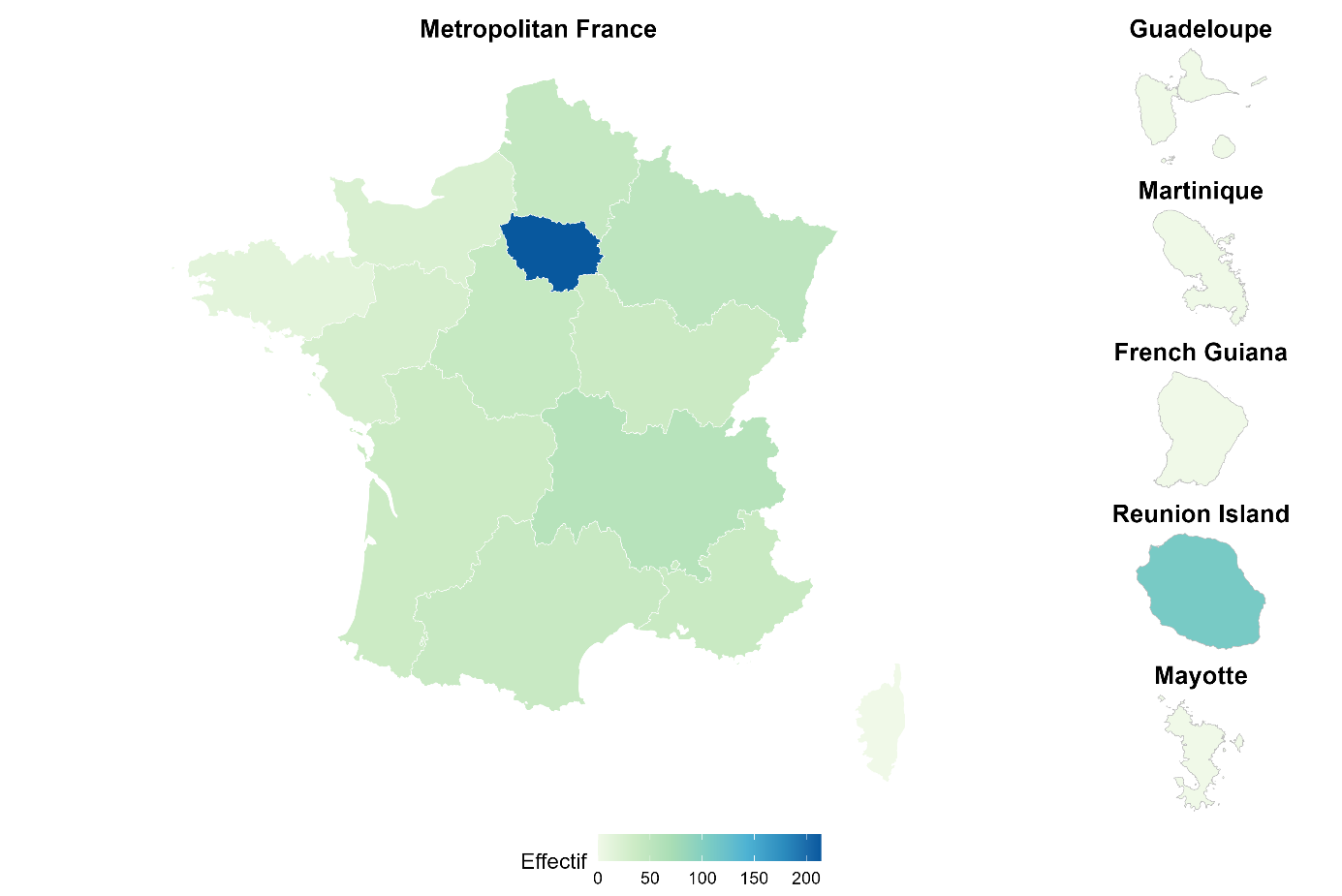
**

**Supplementary eFigure S1. Geographic distribution of respondents across French regions**
Legend: Choropleth map showing the number of survey respondents by French region (metropolitan and overseas). Regions are shaded by respondent count (darker = higher). Regional assignment uses the postal code reported by participants.
Footnotes: Map for illustration; counts correspond to the analytic sample (N=756). Color scale indicates absolute counts, not rates.


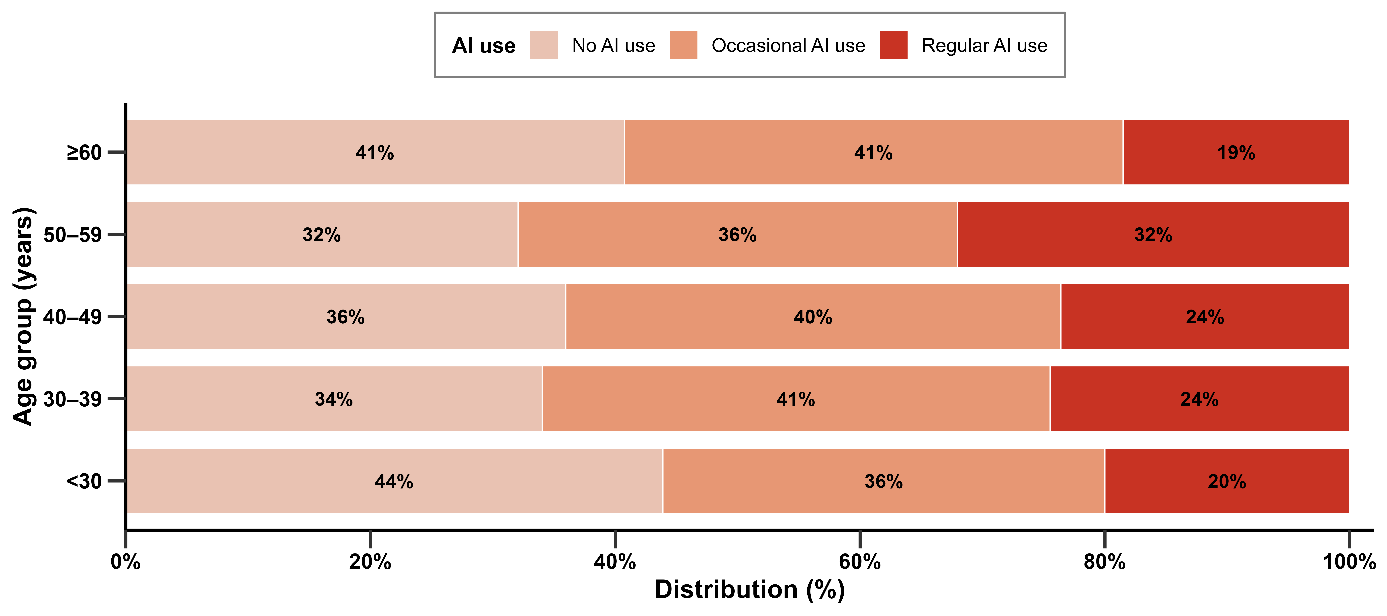


**Supplementary eFigure S2. Distribution of AI use, according to age group.** Shown are the percentages of respondents in each age group who reported no AI use, occasional AI use, or regular AI use. Percentages are displayed within the bars

**
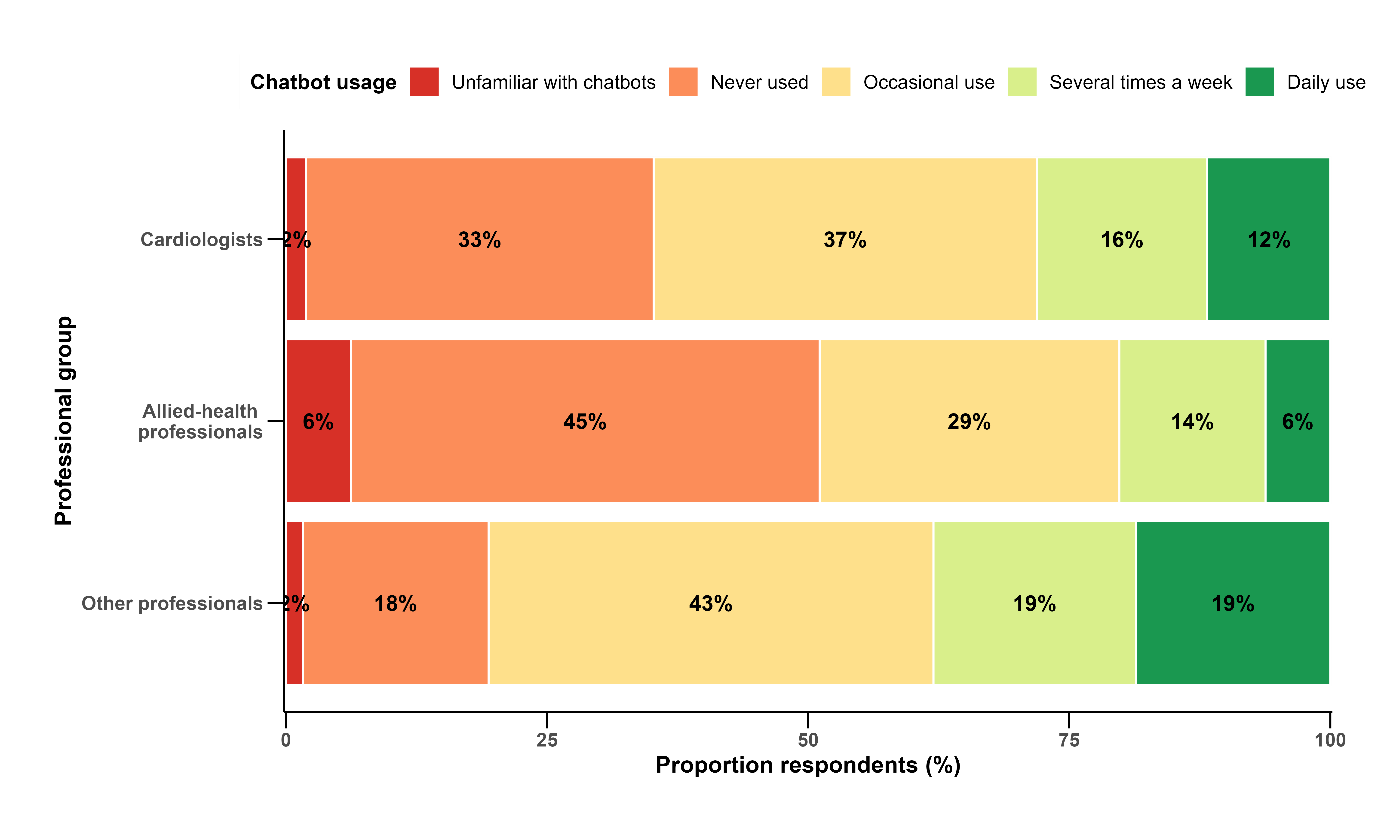
**

**Supplementary eFigure S3. Frequency of chatbot use across professional groups**
Legend: Stacked bars of chatbot use frequency by professional group (cardiologists, allied-health professionals, other professionals). Bars display the proportion within each group for categories ranging from “no use” to “regular use.”
Footnotes: Group comparison by χ² test of independence (overall p as reported in text). Percentages computed with the number of respondents in each group as denominator.


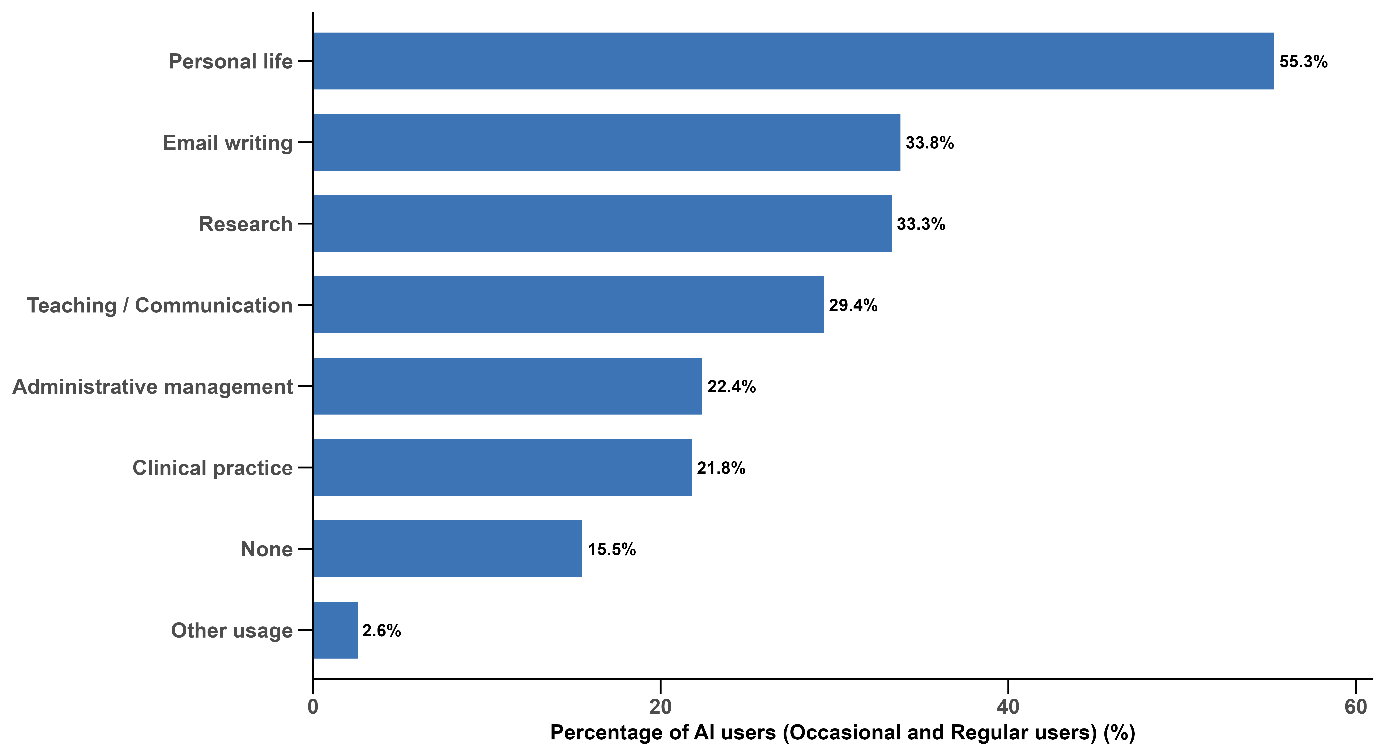


**Supplementary eFigure S4. Domains of chatbot use across professional groups**
Legend: Stacked bars showing the distribution of chatbot use by domain (clinical practice, research, teaching/communication, administrative tasks, personal use, email writing, other) across professional groups.
Footnotes: Multiple-choice question; percentages sum to >100% within groups.

**
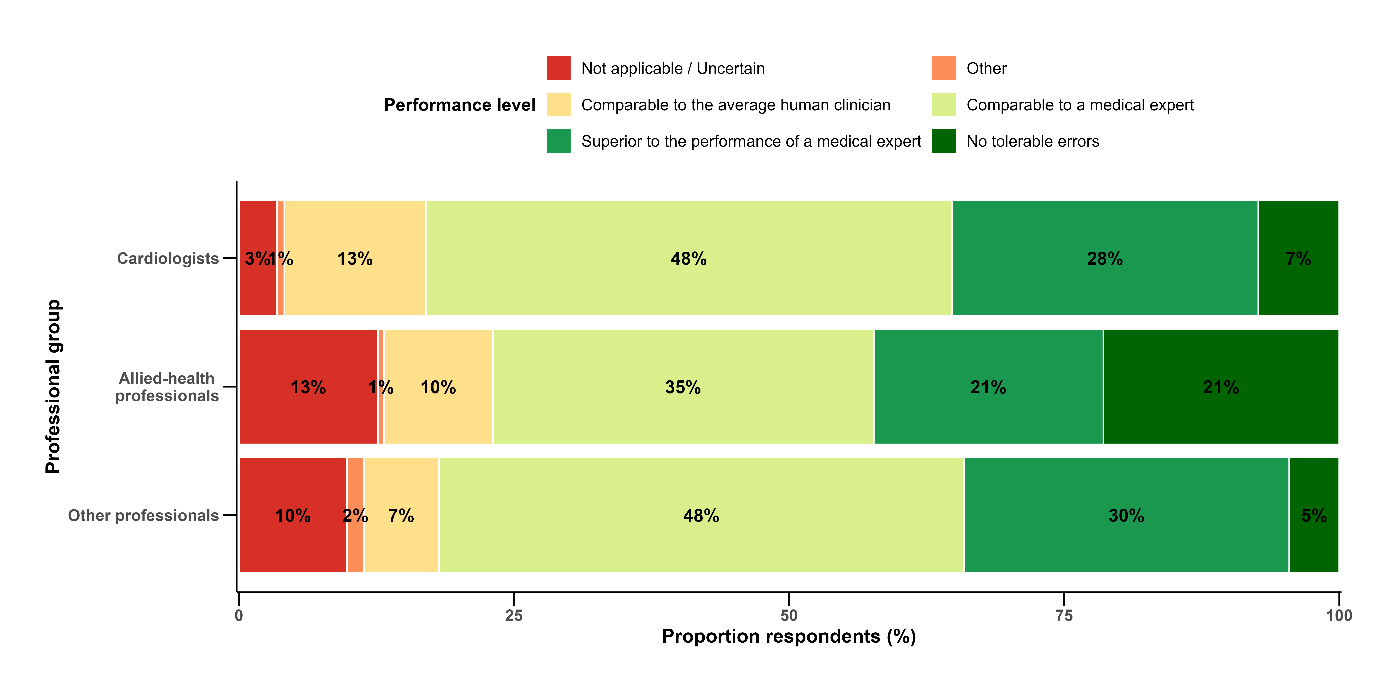
**

**Supplementary eFigure S5. Acceptable performance thresholds for AI across professional groups**
Legend: Stacked bars showing acceptable AI performance thresholds (e.g., “average clinician,” “expert,” “beyond expert,” “not applicable/other”) for each professional group.
Footnotes: Threshold categories derived from a 4-level single-choice item; percentages use group totals as denominators.

## **Supplementary eTables**

**Supplementary eTable S1. CHERRIES checklist (Checklist for Reporting Results of Internet E-Surveys)**

| **Checklist Item** | **Explanation** | **Location in the paper** |
| --- | --- | --- |
| **Survey design** | Target population: cardiology professionals (senior cardiologists, residents, nurses, technicians, engineers, decision-makers). Stratified sampling by professional role, type of institution (academic, public, private) and geographic region to ensure representation. | Methods §“Study design and population” |
| **IRB approval** | Approved by institutional Research Ethics Committee; reference: CER-BDX 2024-250. | Methods §“Ethics” |
| **Informed consent** | Electronic informed consent on first page; participants informed of study purpose, investigator identity, CHU La Réunion as data controller, GDPR compliance, estimated 15-min duration, voluntary participation, data anonymization and storage. | Methods §“Ethics & consent” |
| **Data protection** | Hosted on SKEZIA (certified health data host, GDPR-compliant). Multi-step secure authentication (unique account + email code). Data stored on certified health data servers; investigators had no access to identifiable data. | Methods §“Data protection” |
| **Development & testing** | Questionnaire developed by SFC AI Circle and the College of Cardiologists in Training, reviewed by cardiology & AI experts; pilot-tested for feasibility and technical functionality before launch. | Methods §“Survey development” |
| **Open vs closed survey** | Closed survey: only registered participants with secure account & email confirmation could respond. | Methods §“Recruitment & authentication” |
| **Contact mode** | Initial contact via professional networks and e-mail lists (SFC, CNCH, CNCF). | Methods §“Recruitment strategy” |
| **Advertising the survey** | Announced via: (1) dedicated SFC website page, (2) SKEZIA minisite (progress updates & ambassador ranking), (3) LinkedIn posts, (4) newsletters. Screenshots/wording provided in Supplement. | Methods §“Recruitment strategy”; Supplement |
| **Web/e-mail type** | Web-based e-survey hosted on SKEZIA with automatic response capture. | Methods §“Data collection” |
| **Context** | Survey posted on official SFC channels targeting certified cardiology professionals; not accessible to the general public. The SKEZIA minisite displayed recruitment progress and ambassador rankings. | Methods §“Recruitment strategy” |
| **Mandatory vs voluntary** | Voluntary participation; no obligation to complete for any visitor. | Methods §“Recruitment strategy” |
| **Incentives** | Non-monetary: co-authorship offered to top recruiters; no financial or prize-based incentives. | Methods §“Recruitment strategy” |
| **Time/Date** | Data collected from 4 December 2024 to 1 March 2025. | Methods §“Survey timeline” |
| **Randomisation of items** | No randomisation; fixed order of items. | Methods §“Survey development” |
| **Adaptive questioning** | Yes: branching logic used. All respondents answered a common core (~20 items) for comparability; profession-specific modules added to enhance relevance. | Methods §“Survey development” |
| **Number of items** | Average 35 items per professional group (±1–2 depending on profession). | Methods §“Survey development” |
| **Number of screens** | 7 pages total, including consent page and final thank-you page. | Methods §“Survey development” |
| **Completeness check** | Mandatory fields for key items; automated checks for missing responses; non-response options available (“Prefer not to say”). | Methods §“Data quality control” |
| **Review step** | Respondents could review and change answers using a “Back” button before submission. | Methods §“Data quality control” |
| **Unique visitor definition** | One unique account per participant; unique email authentication prevented duplicate entries. | Methods §“Authentication” |
| **View rate** | Not applicable (closed survey). | NA |
| **Participation rate** | Calculated as participants agreeing to participate after registration / registered users accessing first page. | Results §“Participation metrics” |
| **Completion rate** | Calculated as respondents submitting last page / participants agreeing to participate; reported in Results. | Results §“Participation metrics” |
| **Cookies used** | Not used; secure account authentication sufficient to prevent duplicates. | NA |
| **IP check** | Not used; secure authentication system rendered this unnecessary. | NA |
| **Log-file analysis** | Not used; duplicate prevention via authentication only. | NA |
| **Registration** | Secure account creation required; once submitted, survey locked for that account. | Methods §“Authentication” |
| **Handling incomplete questionnaires** | All analyses were conducted on an available-case basis; the analytic sample size is reported for each table/figure when necessary. | Methods §“Statistical analysis” |
| **Atypical timestamps** | None; no exclusion based on completion time. | Methods §“Statistical analysis” |
| **Statistical correction** | No post-stratification weights were applied; results are unweighted. Potential imbalances from the non-proportional quota design were addressed by prespecified covariate adjustment in multivariable models and subgroup/sensitivity analyses. | Methods §“Statistical analysis” |

This checklist has been modified from Eysenbach G. Improving the quality of Web surveys: the Checklist for Reporting Results of Internet E-Surveys (CHERRIES). J Med Internet Res. 2004 Sep 29;6(3):e34 [erratum in J Med Internet Res. 2012; 14(1): e8.]. Article available at [https://www.jmir.org/2004/3/e34](https://www.jmir.org/2004/3/e34/)/; erratum available <https://www.jmir.org/2012/1/e8/>. Copyright ©Gunther Eysenbach. Originally published in the [Journal of Medical Internet](http://www.jmir.org) Research, 29.9.2004 and 04.01.2012.

**Supplementary eTable S2. Comparison of baseline characteristics between completed and non-completed surveys**

|  | **Completed**  **(n = 756)** | **Non - completed**  **(n = 255)** | **p-value** |
| --- | --- | --- | --- |
| **Demographics** |  |  |  |
| Age, mean ± SD | 39.44 ± 11.88 | 39.26 ± 12.91 | 0.866 |
| Female sex | 353 (46.7) | 73 (47.4) | 0.872 |
| Missing data, n(%) |  | 101 (39.6) |  |
| **Professional categories** |  |  |  |
| Cardiologists | 440 (64.7) | 87 (64) | 0.432 |
| Allied-health professionals† | 184 (27.1) | 42 (30.9) |  |
| AI developer / IT-Engineer | 8 (1.2) | 0 (0) |  |
| Policy / Management stakeholder | 26 (3.8) | 2 (1.5) |  |
| Technician, engineer, or IT manager | 22 (3.2) | 5 (3.7) |  |
| Other professionnals | 76 (10.1) | 0 (0) |  |
| Missing data, n(%) | 0 (0) | 119 (46.7) |  |
| **Workplace** |  |  |  |
| University hospital | 333 (51.3) | 43 (36.1) | 0.002 (**) |
| Non-university hospital / clinic‡ | 237 (36.4) | 56 (47.1) | 0.028 (*) |
| Other | 29 (4.4) | 6 (5) | 0.765 |
| Missing data, n(%) | 105 (13.9) | 136 (53.3) |  |

**Supplementary eTable S3. Sensitivity analysis comparing multivariable logistic regression models with and without age adjustment**

|  | **Regular AI use** | | | |  |
| --- | --- | --- | --- | --- | --- |
|  | | **OR without age (IC95)** | **OR with age (IC95)** | **∆β (%)** | |
| Age (per year) | | NA | 0.97 [0.95; 0.99], p = 0.003 | NA | |
| Female (vs Male) | | 1.57 [1.01; 2.47], p = 0.047 | 1.72 [1.10; 2.72], p = 0.019 | 20 | |
| Prior AI training (vs No) | | 3.26 [1.63; 6.59], p = 0.001 | 3.46 [1.71; 7.10], p = 0.001 | 5 | |
| Cardiologists (vs Allied-health professionals) | | 1.03 [0.62; 1.72], p = 0.904 | 0.99 [0.60; 1.66], p = 0.983 | -117 | |
| University hospital (vs Other) | | 1.04 [0.70; 1.53], p = 0.856 | 0.75 [0.48; 1.16], p = 0.200 | -904 | |
| Active in research (vs No) | | 2.72 [1.77; 4.18], p = 0.000 | 3.15 [2.01; 4.93], p <0.001 | 14 | |

|  | **High Trust in AI Diagnostic** | | |
| --- | --- | --- | --- |
|  | **OR without age (IC95)** | **OR with age (IC95)** | **∆β (%)** |
| Age (per year) | NA | 1.00 [0.98; 1.02], p = 0.959 | NA |
| Female (vs Male) | 1.07 [0.70; 1.63], p = 0.742 | 1.07 [0.70; 1.63], p = 0.750 | -2 |
| Prior AI training (vs No) | 5.86 [2.05; 24.74], p = 0.004 | 5.86 [2.05; 24.74], p = 0.004 | 0.0 |
| Cardiologists (vs Allied-health professionals) | 1.60 [1.02; 2.51], p = 0.039 | 1.60 [1.02; 2.51], p = 0.039 | 0 |
| University hospital (vs Other) | 0.88 [0.61; 1.27], p = 0.493 | 0.88 [0.58; 1.34], p = 0.557 | -4 |
| Active in research (vs No) | 1.16 [0.73; 1.87], p = 0.533 | 1.16 [0.73; 1.88], p = 0.544 | -1 |

|  | **Expect > Expert performance** | | |
| --- | --- | --- | --- |
|  | **OR without age (IC95)** | **OR with age (IC95)** | **∆β (%)** |
| Age (per year) | NA | 1.02 [1.00; 1.04], p = 0.013 | NA |
| Female (vs Male) | 0.89 [0.61; 1.32], p = 0.566 | 0.84 [0.57; 1.24], p = 0.373 | 58 |
| Prior AI training (vs No) | 0.88 [0.44; 1.73], p = 0.717 | 0.84 [0.41; 1.67], p = 0.631 | 34 |
| Cardiologists (vs Allied-health professionals) | 0.61 [0.40; 0.93], p = 0.022 | 0.62 [0.41; 0.96], p = 0.031 | -6 |
| University hospital (vs Other) | 1.03 [0.73; 1.45], p = 0.874 | 1.31 [0.88; 1.95], p = 0.188 | 856 |
| Active in research (vs No) | 1.44 [0.95; 2.19], p = 0.084 | 1.31 [0.85; 2.00], p = 0.215 | -27 |

|  | **Training need** | | |
| --- | --- | --- | --- |
|  | **OR without age (IC95)** | **OR with age (IC95)** | **∆β (%)** |
| Age (per year) | NA | 1.00 [0.97; 1.03], p = 0.943 | NA |
| Female (vs Male) | 1.10 [0.61; 2.01], p = 0.744 | 1.11 [0.60; 2.03], p = 0.739 | 3 |
| Prior AI training (vs No) | 2.55 [0.73; 16.11], p = 0.211 | 2.55 [0.73; 16.13], p = 0.211 | 0 |
| Cardiologists (vs Allied-health professionals) | 2.55 [1.41; 4.66], p = 0.002 | 2.55 [1.40; 4.65], p = 0.002 | -0 |
| University hospital (vs Other) | 0.85 [0.50; 1.42], p = 0.537 | 0.84 [0.48; 1.48], p = 0.553 | 5 |
| Active in research (vs No) | 1.42 [0.71; 3.09], p = 0.343 | 1.43 [0.71; 3.12], p = 0.344 | 1 |

|  | **Social influence** | | |
| --- | --- | --- | --- |
|  | **OR without age (IC95)** | **OR with age (IC95)** | **∆β (%)** |
| Age (per year) | NA | 0.98 [0.96; 1.00], p = 0.066 | NA |
| Female (vs Male) | 0.42 [0.24; 0.72], p = 0.002 | 0.44 [0.25; 0.76], p = 0.004 | -6.1 |
| Prior AI training (vs No) | 1.09 [0.48; 2.78], p = 0.853 | 1.11 [0.49; 2.85], p = 0.812 | 28.8 |
| Cardiologists (vs Allied-health professionals) | 2.53 [1.43; 4.50], p = 0.001 | 2.50 [1.41; 4.47], p = 0.002 | -1.4 |
| University hospital (vs Other) | 1.07 [0.68; 1.67], p = 0.780 | 0.87 [0.53; 1.44], p = 0.596 | -312.3 |
| Active in research (vs No) | 0.65 [0.39; 1.10], p = 0.099 | 0.70 [0.42; 1.20], p = 0.186 | -18.4 |

Note : We assessed the potential confounding effect of age by comparing two nested logistic regression models for each outcome (unadjusted vs adjusted for age). Overall, the effect estimates were stable, with very similar ORs and 95% CIs across models, suggesting limited confounding by age. The only outcome showing a more noticeable sensitivity to age adjustment was “Expect > Expert performance.” The most extreme relative changes in coefficients (Δβ%) occurred when the initial coefficients were close to zero (i.e., OR≈1), a situation in which percent change metrics can be inflated despite minimal absolute change. Given its clinical relevance and to ensure comparability across models, we retained age as an adjustment variable in all final analyses.

**Supplementary eTable S4. Sociodemographic characteristics, training and attitudes by age group**

|  | **<30**  **(n = 181)** | **30–39**  **(n = 243)** | **40–49**  **(n = 186)** | **50–59**  **(n = 86)** | **≥60**  **(n = 60)** | **p-value** |
| --- | --- | --- | --- | --- | --- | --- |
| **Demographics** |  |  |  |  |  |  |
| Female sex | 90 (49.7) | 123 (50.6) | 99 (53.2) | 31 (36) | 10 (16.7) | p < 0.001 (***) |
| **Professional categories** |  |  |  |  |  |  |
| Cardiologists | 110 (60.8) | 153 (63) | 80 (43) | 50 (58.1) | 47 (78.3) | p < 0.001 (***) |
| Allied-health professionals† | 38 (21) | 63 (25.9) | 58 (31.2) | 22 (25.6) | 3 (5) |  |
| Other professionnals | 33 (18.2) | 27 (11.1) | 48 (25.8) | 14 (16.3) | 10 (16.7) |  |
| **Workplace** |  |  |  |  |  |  |
| University hospital vs others workplace | 152 (84.4) | 112 (46.1) | 75 (40.3) | 26 (30.2) | 11 (18.3) | p < 0.001 (***) |
| **Training & attitudes** |  |  |  |  |  |  |
| Formal AI training | 13 (7.2) | 22 (9.1) | 11 (6.2) | 8 (10.3) | 3 (5.6) | 0.689 |
| No perceived need for training | 24 (13.3) | 31 (12.9) | 18 (9.7) | 12 (14) | 6 (10.2) | 0.765 |
| Missing data, n(%) | 0 (0) | 3 (1.2) | 1 (0.5) | 0 (0) | 1 (1.7) |  |
| Would adopt if colleagues succeed (Yes) | 160 (88.9) | 210 (86.4) | 155 (83.8) | 64 (75.3) | 49 (81.7) | 0.058 |
| Missing data, n(%) | 0 (0) | 1 (0.5) | 1 (1.2) | 0 (0) | 0 (0) |  |
| Expected performance (Expert-level performance) | 40 (23.7) | 100 (43.1) | 79 (48.8) | 30 (41.7) | 26 (45.6) | p < 0.001 (***) |
| Research activity | 12 (6.6) | 11 (4.5) | 24 (12.9) | 14 (16.3) | 3 (5) | 0.022 (*) |

**Supplementary eTable S5.** Cardiologists’ subspecialties and AI attitudes

|  | **Cardiologist (n = 429)** | **No AI use(n = 132)** | **Occasional AI use(n = 193)** | **Regular AI use(n = 104)** | **p-value** |
| --- | --- | --- | --- | --- | --- |
| **Sub-specialty (réponses multiples)** |  |  |  |  |  |
| General cardiology | 178 (41.5%) | 62 (47%) | 74 (38.3%) | 42 (40.4%) | p = 0.290 |
| Heart failure and cardiomyopathies | 88 (20.5%) | 20 (15.2%) | 39 (20.2%) | 29 (27.9%) | p = 0.055 |
| Cardiac intensive care unit (CICU) | 64 (14.9%) | 16 (12.1%) | 30 (15.5%) | 18 (17.3%) | p = 0.511 |
| Congenital and pediatric cardiology | 26 (6.1%) | 10 (7.6%) | 14 (7.3%) | 2 (1.9%) | p = 0.126 |
| Cardiac electrophysiology | 77 (17.9%) | 18 (13.6%) | 33 (17.1%) | 26 (25%) | p = 0.072 |
| Interventional cardiology | 93 (21.7%) | 23 (17.4%) | 47 (24.4%) | 23 (22.1%) | p = 0.328 |
| Cardiac imaging | 86 (20%) | 12 (9.1%) | 45 (23.3%) | 29 (27.9%) | p < 0.001 |
| Cardiac rehabilitation | 33 (7.7%) | 13 (9.8%) | 17 (8.8%) | 3 (2.9%) | p = 0.101 |
| Cardiovascular prevention and hypertension | 33 (7.7%) | 11 (8.3%) | 15 (7.8%) | 7 (6.7%) | p = 0.899 |
| Cardio-oncology | 21 (4.9%) | 2 (1.5%) | 12 (6.2%) | 7 (6.7%) | p = 0.095 |
| Other specialities | 11 (2.6%) | 2 (1.5%) | 3 (1.6%) | 6 (5.8%) | p = 0.097 |
| No subspecialty | 38 (8.9%) | 22 (16.7%) | 14 (7.3%) | 2 (1.9%) | p < 0.001 |
| **Career stage** |  |  |  |  |  |
| Resident / Fellow | 136 (31.8%) | 54 (41.2%) | 55 (28.5%) | 27 (26%) | p = 0.064 |
| Early-career cardiologist | 28 (6.5%) | 8 (6.1%) | 11 (5.7%) | 9 (8.7%) |  |
| Senior cardiologist | 264 (61.7%) | 69 (52.7%) | 127 (65.8%) | 68 (65.4%) |  |
| Missing data, n(%) | 1 (0.2%) | 1 (0.8%) | - | - |  |

Legend: Distribution of cardiology subspecialties (multiple responses) and career stage in cardiologists, stratified by AI-use category (no/occasional/regular).
Footnotes: Multiple-choice for subspecialties; row percentages may exceed 100%. Counts reflect available answers for AI-use status; cardiologists with missing AI-use were excluded from this stratified table. p-values from χ² or Fisher’s exact tests as appropriate. Abbreviations: CICU, cardiac intensive care unit.

**Supplementary eTable S6.** Allied-health professional roles and AI attitudes

|  | **Allied-health professionals (n=176)** | **No AI use(n = 101)** | **Occasional AI use(n = 46)** | **Regular AI use(n = 29)** | **p-value** |
| --- | --- | --- | --- | --- | --- |
| **Subspecialty (multiple answers)** |  |  |  |  |  |
| General cardiology | 87 (49.4%) | 57 (56.4%) | 16 (34.8%) | 14 (48.3%) | p = 0.051 |
| Heart failure and cardiomyopathies | 69 (39.2%) | 34 (33.7%) | 18 (39.1%) | 17 (58.6%) | p = 0.053 |
| Cardiac intensive care unit (CICU) | 71 (40.3%) | 49 (48.5%) | 13 (28.3%) | 9 (31%) | p < 0.05 |
| Congenital and pediatric cardiology | 11 (6.2%) | 6 (5.9%) | 3 (6.5%) | 2 (6.9%) | p = 1.000 |
| Cardiac electrophysiology | 74 (42%) | 36 (35.6%) | 24 (52.2%) | 14 (48.3%) | p = 0.129 |
| Interventional cardiology | 72 (40.9%) | 40 (39.6%) | 21 (45.7%) | 11 (37.9%) | p = 0.739 |
| Cardiac imaging | 13 (7.4%) | 3 (3%) | 6 (13%) | 4 (13.8%) | p < 0.05 |
| Cardiac rehabilitation | 8 (4.5%) | 4 (4%) | 3 (6.5%) | 1 (3.4%) | p = 0.878 |
| Cardiovascular prevention and hypertension | 23 (13.1%) | 11 (10.9%) | 7 (15.2%) | 5 (17.2%) | p = 0.591 |
| Cardio-oncology | 6 (3.4%) | 2 (2%) | 1 (2.2%) | 3 (10.3%) | p = 0.115 |
| **Allied-health roles** |  |  |  |  |  |
| Nurse manager | 9 (5.1%) | 4 (4%) | 5 (10.9%) | 0 (0%) | p < 0.05 |
| General cardiology care nurse | 90 (51.1%) | 60 (59.4%) | 15 (32.6%) | 15 (51.7%) |  |
| Cath-lab nurse / Electroradiology technologist | 27 (15.3%) | 13 (12.9%) | 10 (21.7%) | 4 (13.8%) |  |
| Advanced practice nurse – HF / Echocardiography technician | 18 (10.2%) | 6 (5.9%) | 6 (13%) | 6 (20.7%) |  |
| Other allied-health qualification | 32 (18.2%) | 18 (17.8%) | 10 (21.7%) | 4 (13.8%) |  |

Legend: Subspecialty areas (multiple responses) and role/position among allied-health professionals, stratified by AI-use category.
Footnotes: Multiple-choice for subspecialties and roles. Counts reflect available answers for AI-use status; allied-health respondents with missing AI-use were excluded from this stratified table. p-values from χ² or Fisher’s exact tests as appropriate.

**Supplementary eTable S7.** Reported use of AI tools by professional group and social influence according to professional group (detailed counts)

|  | **Overall (n=756)** | **Cardiologists (n=440)** | **Allied-health professionals (n=184)** | **Other professionals (n=132)** | **p-value** |
| --- | --- | --- | --- | --- | --- |
| **Use of AI tools in cardiology** |  |  |  |  |  |
| AI-assisted image analysis | 236 (32.4%) | 199 (46.6%) | 21 (11.9%) | 16 (12.8%) | p < 0.001 |
| Clinical predictive modeling | 63 (8.6%) | 50 (11.7%) | 2 (1.1%) | 11 (8.8%) | p < 0.001 |
| Medical data processing | 77 (10.6%) | 50 (11.7%) | 13 (7.3%) | 14 (11.2%) | p = 0.274 |
| Patient monitoring and management with AI | 128 (17.6%) | 78 (18.3%) | 38 (21.5%) | 12 (9.6%) | p < 0.05 |
| Medical diagnostic support system | 65 (8.9%) | 31 (7.3%) | 10 (5.6%) | 24 (19.2%) | p < 0.001 |
| Personalization of care via AI | 22 (3%) | 14 (3.3%) | 4 (2.3%) | 4 (3.2%) | p = 0.794 |
| Other | 41 (5.6%) | 22 (5.2%) | 7 (4%) | 12 (9.6%) | p = 0.089 |
| No use | 320 (43.9%) | 143 (33.5%) | 108 (61%) | 69 (55.2%) | p < 0.001 |
| Missing data | 27 (3.6%) | 13 (3%) | 7 (3.8%) | 7 (5.3%) |  |
| **Social influence** |  |  |  |  |  |
| No, I am a pioneer in adopting it | 86 (11.4) | 54 (12.3) | 19 (10.3) | 13 (9.9) | p < 0.001 (***) |
| No, I am skeptical about AI | 29 (3.9) | 8 (1.8) | 15 (8.2) | 6 (4.6) |  |
| Yes, if my colleagues use it successfully | 185 (24.6) | 128 (29.2) | 30 (16.3) | 27 (20.6) |  |
| Yes, if it is validated by clinical guidelines or scientific studies | 453 (60.2) | 248 (56.6) | 120 (65.2) | 85 (64.9) |  |
| Missing data | 3 (0.4) | 2 (0.5) |  | 1 (0.8) |  |
|  |  |  |  |  |  |

Legend: Frequencies of specific AI tools used (e.g., image analysis, predictive modelling, monitoring/management, diagnostic support, personalization) by professional group.
Footnotes: Multiple-choice; respondents could select several tools. “No use” indicates none of the tool categories selected. Group comparison by χ²/Fisher’s exact. Abbreviations: AI, artificial intelligence.

**Supplementary eTable S8.** Detailed chatbot use by professional group and domain

|  | **Overall (n=756)** | **Cardiologists (n=440)** | **Allied-health professionals (n=184)** | **Other professionals (n=132)** | **p-value** |
| --- | --- | --- | --- | --- | --- |
| Clinical practice | 115 (15.7%) | 91 (21.3%) | 13 (7.3%) | 11 (8.5%) | p < 0.001 |
| Research | 186 (25.3%) | 107 (25%) | 35 (19.8%) | 44 (34.1%) | p < 0.05 |
| Teaching / Communication | 165 (22.5%) | 100 (23.4%) | 25 (14.1%) | 40 (31%) | p < 0.01 |
| Administrative management | 123 (16.8%) | 60 (14%) | 23 (13%) | 40 (31%) | p < 0.001 |
| Personal life | 356 (48.5%) | 204 (47.7%) | 79 (44.6%) | 73 (56.6%) | p = 0.102 |
| Writing e-mails | 206 (28.1%) | 120 (28%) | 34 (19.2%) | 52 (40.3%) | p < 0.001 |
| Others | 16 (2.2%) | 4 (0.9%) | 7 (4%) | 5 (3.9%) | p < 0.05 |
| No use AI tools | 225 (30.7%) | 129 (30.1%) | 74 (41.8%) | 22 (17.1%) | p < 0.001 |
| Missing data | 22 (2.9%) | 12 (2.7%) | 7 (3.8%) | 3 (2.3%) |  |

Legend: Counts and percentages of chatbot use across domains (clinical practice, research, teaching/communication, administrative, personal, email writing, other) by professional group.
Footnotes: Multiple-choice; percentages may exceed 100% within groups. “No use AI tools” corresponds to respondents reporting no chatbot use. Group comparison by χ²/Fisher’s exact.

**Supplementary eTable S9.** AI training needs and preferences by professional group

|  | **Overall (n=756)** |  | **Cardiologists (n=440)** | **Allied-health professionals (n=184)** | **Other professionals (n=132)** | **p-value** |
| --- | --- | --- | --- | --- | --- | --- |
| **AI training needs** |  |  |  |  |  |  |
| Hands-on workshops or face-to-face courses | 402 (53.5%) |  | 245 (56.1%) | 103 (56.3%) | 54 (41.2%) | p < 0.01 |
| Hands-on videos | 374 (49.8%) |  | 233 (53.3%) | 85 (46.4%) | 56 (42.7%) | p = 0.061 |
| Online courses / MOOC | 333 (44.3%) |  | 212 (48.5%) | 66 (36.1%) | 55 (42%) | p < 0.05 |
| CPD training | 190 (25.3%) |  | 128 (29.3%) | 35 (19.1%) | 27 (20.6%) | p < 0.05 |
| Conferences | 194 (25.8%) |  | 124 (28.4%) | 43 (23.5%) | 27 (20.6%) | p = 0.145 |
| Scientific articles | 161 (21.4%) |  | 102 (23.3%) | 26 (14.2%) | 33 (25.2%) | p < 0.05 |
| Mentoring by a trained colleague | 214 (28.5%) |  | 145 (33.2%) | 38 (20.8%) | 31 (23.7%) | p < 0.01 |
| Other formations | 5 (0.7%) |  | 1 (0.2%) | 2 (1.1%) | 2 (1.5%) | p = 0.119 |
| No training needs | 91 (12.1%) |  | 35 (8%) | 34 (18.6%) | 22 (16.8%) | p < 0.001 |
| Missing data | 5 (0.7%) |  | 3 (0.7%) | 1 (0.5%) | 1 (0.8%) |  |

Legend: Training formats selected (hands-on workshops, videos, MOOCs, CPD, conferences, scientific articles, mentoring, other, none) by professional group.
Footnotes: Multiple-choice; respondents could select several formats. “No training needs” is a single-choice option distinct from other selections. Group comparison by χ²/Fisher’s exact. Abbreviations: MOOC, massive open online course; CPD, continuing professional development.

**Supplementary eTable S10.** Multivariable logistic regression models for AI adoption and perceptions

|  | **Expect ≥ Expert performance** | **High Trust in AI Diagnostic** | **Regular AI use** | **Social influence** | **Training need** |
| --- | --- | --- | --- | --- | --- |
|  | aOR (IC95%), p-value | aOR (IC95%), p-value | aOR (IC95%), p-value | aOR (IC95%), p-value | aOR (IC95%), p-value |
| Age > 40 years (vs ≤40) | 1.32 (0.89 – 1.95), p=0.169 | 0.96 (0.63 – 1.46), p=0.847 | **0.62 (0.40 – 0.96), p=0.035** | **0.52 (0.32 – 0.84), p=0.008** | 1.37 (0.76 – 2.50), p=0.301 |
| Men (vs. Women) | 0.87 (0.59 – 1.29), p=0.493 | 1.08 (0.70 – 1.64), p=0.730 | **1.64 (1.05 – 2.59), p=0.031** | **0.44 (0.25 – 0.76), p=0.004** | 1.07 (0.59 – 1.96), p=0.816 |
| Prior AI Training (vs no) | 0.88 (0.43 – 1.73), p=0.713 | **5.85 (2.04 – 24.69), p=0.004** | **3.22 (1.60 – 6.55), p=0.001** | 1.06 (0.47 – 2.72), p=0.901 | 2.56 (0.74 – 16.21), p=0.208 |
| Cardiologists (vs Allied-health professionals) | **0.62 (0.41 – 0.96), p=0.030** | **1.59 (1.02 – 2.50), p=0.042** | 0.98 (0.59 – 1.65), p=0.953 | **2.37 (1.33 – 4.26), p=0.003** | **2.64 (1.45 – 4.85), p=0.002** |
| University hospital (vs other) | 1.14 (0.78 – 1.66), p=0.501 | 0.87 (0.58 – 1.29), p=0.480 | 0.88 (0.58 – 1.34), p=0.562 | 0.85 (0.52 – 1.38), p=0.514 | 0.94 (0.54 – 1.62), p=0.816 |
| Active in research (vs no) | 1.38 (0.91 – 2.11), p=0.133 | 1.17 (0.73 – 1.89), p=0.520 | **2.94 (1.90 – 4.58), p<0.001** | 0.72 (0.43 – 1.24), p=0.225 | 1.35 (0.67 – 2.95), p=0.421 |

Legend: Adjusted odds ratios (aOR) with 95% CIs for five binary outcomes: (1) expectation of AI performance > expert, (2) high trust in diagnostic AI, (3) regular AI use, (4) adoption under social influence, (5) self-reported training need.
Footnotes: Covariates included in all models a priori: prior AI training (no [ref] vs yes), professional group (allied-health [ref] vs cardiologists), sex (women [ref] vs men), age (≤40 [ref] vs >40 years), practice setting (other [ref] vs university hospital), active in research (no [ref] vs yes). Outcomes were coded as binary variables as defined in Methods. aORs from logistic regression; 95% CI in parentheses. Abbreviations: aOR, adjusted odds ratio; CI, confidence interval.

**Supplementary eTable S11.** Ambassadors of the study

(content hidden for blinded peer review)

*This table lists the regional and institutional ambassadors who contributed to survey dissemination. In compliance with the blinded peer-review process, individual names and institutions have been temporarily removed. The complete version will be made available upon acceptance of the manuscript.*

.
