# Supplementary material for: Artificial intelligence adoption in French cardiovascular care: a multiprofessional survey of barriers and facilitators
Source: Eur Heart J Digit Health. 2026 Apr 17;7(4):ztag042. doi: 10.1093/ehjdh/ztag042 (PMC13131982; doi:10.1093/ehjdh/ztag042)
Supplement: ztag042_Supplementary_Data [file ztag042_supplementary_data.zip › Revised_Supplementary_Not_For_Review.docx]

# Supplemental Material

**Editorial note (for editors only)**

This table lists the regional and institutional ambassadors who contributed to the dissemination of the INSIGHT-AI France survey.
In accordance with the *blinded peer review policy*, this document is submitted as a **“Supplemental File Not For Review”** and will not be shared with reviewers.

**Supplementary eTable S11.** Ambassadors of the study

| Name | Center |
| --- | --- |
| Aboueddahab Chaimae | Department of Cardiovascular Medicine, Nouvel Hôpital Civil, Strasbourg University Hospital, Strasbourg, Cardiology Department, Ibn Sina University Hospital, Mohammed V University of Rabat, Rabat, Morocco |
| Algalarrondo Vincent | AP-HP Nord — Hôpital Bichat, Department of Cardiology, Paris |
| Amara Walid | Rhythmology Unit, Intermunicipal Hospital Group Le Raincy Montfermeil, Montfermeil |
| Bailly Guillaume | Cardiology Department, Saint-Antoine and Tenon Hospitals, AP-HP, Sorbonne University, Paris, ⁹ Sorbonne University, AP-HP, Inserm |
| Baudry Guillaume | Université de Lorraine, Inserm CIC-1433, Inserm U1116, Nancy University Hospital, Nancy; INI-CRCT (Cardiovascular and Renal Clinical Trialists), F-CRIN Network, Nancy |
| Beuque Gauthier | Cardiology Department, Bordeaux University Hospital, Bordeaux |
| Cabaye Nicolas | Cardiology Department, Rouen University Hospital, Rouen |
| Chenard Pierre | Cardiology Department, Limoges University Hospital, Limoges |
| De Saint Nicolas Thomas | Cardiology Department, Lille University Hospital, Lille |
| De Saunière Olivier | Cardiology Practice (CARIC), Riorges (France) |
| Delmas Clément | Intensive Cardiac Care Unit, Cardiology Department, Rangueil University Hospital, Toulouse, France. |
| Deschamps Pauline | Mulhouse Sud-Alsace Regional Hospital Group (GHR), Mulhouse (France |
| Djalakhan Mariama Diallo | Guadeloupe University Hospital — Cardiology Department, Pointe-à-Pitre/Les Abymes (France |
| Domart Séverine | Cardiology Department, University Hospital of La Réunion, Saint-Denis, France |
| El Blidi-Rahmani Manel | AP-HP — Bicêtre Hospital, Cardiology Department, Le Kremlin-Bicêtre (France) |
| Elidrissi Anis | Cardiology Department, Strasbourg University Hospital, Strasbourg |
| Fischer Quentin | Cardiology Practice, Paris |
| Florence Jérémy | Cardiology Department, Clermond-Ferrand University Hospital, Strasbourg |
| Garban Thierry | National Union of Cardiologists, Paris |
| Hascoet Sébastien | Department of Congenital Heart Diseases, Marie Lannelongue Hospital, M3C Network, Le Plessis-Robinson, France |
| Hudelo Julien | Cardiology Department, Amiens University Hospital, Amiens |
| Jourdain Patrick | Ramsay Santé — Research and Academic Affairs Department (Paris, France) |
| Lakhal Youssef | Cardiology Department, University Hospital of La Réunion, Saint-Denis, France |
| Laure Christophe | Clinical Research and Innovation Directorate, Chartres Hospital Center, Le Coudray |
| Lequeux Benoît | Cardiology Department, University Hospital of Poitiers, Poitiers |
| Lucain Paul | Cardiology Department, Amiens University Hospital, Amiens |
| Mette Carole | Centre Cardiologique du Nord, Saint-Denis, 93 |
| Montant Patrick | Alpes-Léman Hospital — Cardiology Department (Contamine-sur-Arve/Annemasse, France) |
| Mrabet Soundous | Cardiology Department, Dijon University Hospital, Dijon, France |
| Perrard Louis | Cardiology Department, Amiens University Hospital, Amiens |
| Régnier Philippe | Hôpital Privé Paul d’Égine — Cardiologie (Champigny-sur-Marne). |
| Reisberg Johann | AP-HP — Bicêtre Hospital, Cardiology Department, Le Kremlin-Bicêtre (France) |
| Si Moussi Thiziri | Clinique du Millénaire, Montpellier |
| Singh Manveer | AP-HP — Hôpital Lariboisière, Service de cardiologie (Paris). |
| Touré Massiré | Institut de Cardiologie d’Abidjan (Côte d’Ivoire). |
| Viscogliosi Simon | Department of Cardiology, Hospices Civils de Lyon, Lyon, France |
| Zebrowski Pierre-André | Cardioparc (Lyon, France) |

Legend: List of regional “ambassadors” who facilitated survey dissemination, with affiliated centres and cities.
Footnotes: Affiliations as provided by contributors; alphabetical order by family name.
